# Dmrt2 promotes transition of endochondral bone formation by linking Sox9 and Runx2

**DOI:** 10.1038/s42003-021-01848-1

**Published:** 2021-03-11

**Authors:** Koichiro Ono, Kenji Hata, Eriko Nakamura, Shota Ishihara, Sachi Kobayashi, Masako Nakanishi, Michiko Yoshida, Yoshifumi Takahata, Tomohiko Murakami, Seiichi Takenoshita, Toshihisa Komori, Riko Nishimura, Toshiyuki Yoneda

**Affiliations:** 1 Department of Molecular and Cellular Biochemistry, Osaka University Graduate School of Dentistry, Osaka, Japan; 2 Department of Orthopedics, Nippon Medical School, Tokyo, Japan; 3 Department of Pathology, Wakayama Medical University, Wakayama, Japan; 4 Advanced Clinical Research Center, Fukushima Medical University, Fukushima, Japan; 5 Department of Cell Biology, Nagasaki University Graduate School of Biomedical Sciences, Nagasaki, Japan

**Keywords:** Cartilage development, Differentiation, Transcription

## Abstract

Endochondral bone formation is fundamental for skeletal development. During this process, chondrocytes undergo multiple steps of differentiation and coordinated transition from a proliferating to a hypertrophic stage, which is critical to advance skeletal development. Here, we identified the transcription factor *Dmrt2* (double-sex and mab-3 related transcription factor 2) as a Sox9-inducible gene that promotes chondrocyte hypertrophy in pre-hypertrophic chondrocytes. Epigenetic analysis further demonstrated that Sox9 regulates *Dmrt2* expression through an active enhancer located 18 kb upstream of the *Dmrt2* gene and that this enhancer’s chromatin status is progressively activated through chondrocyte differentiation. *Dmrt2*-knockout mice exhibited a dwarf phenotype with delayed initiation of chondrocyte hypertrophy. Dmrt2 augmented hypertrophic chondrocyte gene expression including *Ihh* through physical and functional interaction with Runx2. Furthermore, Dmrt2 deficiency reduced Runx2-dependent *Ihh* expression. Our findings suggest that Dmrt2 is critical for sequential chondrocyte differentiation during endochondral bone formation and coordinates the transcriptional network between Sox9 and Runx2.

## Abstract (web summary)

Koichiro Ono et al. report that Dmrt2 is a critical transcription factor for chondrocyte maturation during endochondral ossification. The study shows that Dmrt2 is the direct target of Sox9 and acts as a transcriptional coactivator of Runx2 providing novel insights into the transcription factor network during skeletal development.

## Introduction

Endochondral bone formation is the fundamental process of skeletal development in vertebrates^1^. Most of the mammalian skeleton is formed through this process, and mutations affecting endochondral bone formation cause skeletal abnormalities including chondrodysplasia, characterized by dwarfism and craniofacial abnormalities. Thus, uncovering the mechanism of endochondral bone formation should shed light on the pathophysiology of genetic skeletal disorders.

Endochondral bone formation occurs by sequential steps of chondrocyte differentiation^2^. Chondrocytes first arise from mesenchymal cells derived from cranial neural crest cells, sclerotomes, and lateral plate mesoderm. These cells undergo mesenchymal cell condensation and differentiate into early-stage chondrocytes, including round and proliferating chondrocytes that produce abundant chondrocyte-specific extracellular matrix proteins such as collagen type II alpha 1 chain (Col2a1) and Aggrecan (Acan)^2^. Proliferating chondrocytes then stop proliferating and enlarge their cell size to become pre-hypertrophic and hypertrophic chondrocytes^3^, which are characterized by the expression of *Indian hedgehog* (*Ihh*) and *collagen type X alpha 1 chain* (*Col10a1*), respectively^4,5^. These late-stage chondrocytes then undergo terminal differentiation and produce matrix metalloproteinase 13 (MMP13), which allows vascular invasion into cartilage^6^. Finally, terminal chondrocytes become apoptotic and are replaced by bone^2^. The sequential processes of early, late, and terminal chondrocyte differentiation construct a well-arranged columnar layer of chondrocytes called growth plate chondrocytes.

The function of stage-specific chondrocytes is strictly controlled by critical transcription factors^7–9^. In particular, Sry-related HMG-Box gene 9 (Sox9) plays indispensable roles in chondrocyte development and endochondral bone formation^10^, and runt-related transcription factor 2 (Runx2) and Runx3 play essential roles in chondrocyte hypertrophy by directly regulating *Ihh* expression^9^. Chondrocyte-specific *Sox9*-knockout mice display severe defects in skeletal development and abnormal craniofacial development^10,11^ and mice with double knockout of *Runx2* and *Runx3* show the complete absence of hypertrophic chondrocytes^9^. Notably, Sox9 regulates the expression of early-chondrocyte genes, *Col2a1* and *Acan*, in collaboration with Sox5 and Sox6^12^. Recent genome-wide analyses further demonstrated the genome-wide cooperation of Sox5/6/9 through super-enhancers of chondrocyte genes^12–14^. Because Sox9 expression initiates and promotes *Sox5* and *Sox6* expression^10^, Sox5 and Sox6 are not essential for the initiation of chondrogenesis but are required for the promotion of Sox9-regulated chondrogenesis.

Although it has long been accepted that Sox9 regulates early chondrogenesis, recent studies established that Sox9 is important for chondrocyte hypertrophy. Sox9, in association with myocyte enhancer factor 2C (Mef2c) and AP-1 family members, directly activates *Col10a1* expression to promote chondrocyte hypertrophy^15,16^. Moreover, Sox9 is expressed in upper hypertrophic chondrocytes and maintains *Runx2* expression^15^. These reports strongly indicate that Sox9 target genes are also involved in chondrocyte hypertrophy and unknown molecules mediate the transition from proliferating to hypertrophic chondrocytes and conduct transcriptional machinery of the Sox9–Runx2 axis. However, in contrast to the wealth of knowledge regarding the Sox9 target genes in early chondrogenesis, the roles of Sox9 target genes in chondrocyte hypertrophy remain poorly understood. Thus, uncovering the target genes of Sox9 and their functional roles in chondrocyte hypertrophy would deepen our understanding of endochondral bone formation.

The DMRT (doublesex and mab-3-related transcription factor) family genes are transcription factors that contain a highly conserved DNA binding domain called the DM domain. There are seven Dmrt genes (Dmrt1 to Dmrt7) in mice and humans, which have been shown to play critical roles in sexual regulation, including sex differentiation, sexual dimorphism, and spermatogenesis^17,18^. In addition to sexual regulation, Dmrt genes regulate multiple developmental processes, including neural development, myogenesis, and skeletal development during embryogenesis^19^. Emerging evidence also suggests that Dmrt2 is involved in skeletal development in humans and mice. For example, *Dmrt2*-deficient mice were reported to show various skeletal abnormalities including severe rib malformations, and disorganized ossification of vertebrae and sternum^20^. Furthermore, in humans, a homozygous start-loss variant in *DMRT2* (c.1 A > T;p.[Met1]) shows a different subtype of spondylocostal dysostosis characterized by severe rib anomalies without segmentation defects, short stature (−2 SD), scoliosis, and reduced size of the thoracic cage, which cause respiratory impairment^21^. Moreover, patients with a 9p deletion that removes *DMRT1* and *DMRT2* show severe growth retardation^22^. These reports indicate that Dmrt2 regulates skeletal development, but no studies have investigated its role in endochondral bone formation.

In this study, we discovered that Dmrt2 coordinates endochondral bone formation as a molecule downstream of Sox9. Sox9 and its partners Sox5 and Sox6 increased *Drmt2* gene expression along with chondrocyte differentiation. *Drmt2*-deficient mice showed the dwarf phenotype and delayed endochondral bone formation. Mechanistically, Dmrt2 promoted *Ihh* expression and chondrocyte hypertrophy through physical and functional collaboration with Runx2. The epigenetic analysis further revealed that Sox9 directly bound to H3K27ac positive enhancer located 18 kb upstream of the *Dmrt2* transcriptional start site (TSS) and that the chromatin status of this enhancer became progressively more active through chondrocyte differentiation. Thus, our findings provide novel insights into the transcription network controlling endochondral bone formation.

## Results

### Dmrt2 is a target gene of Sox5/6/9 in primary chondrocytes

**Fig. 1:**
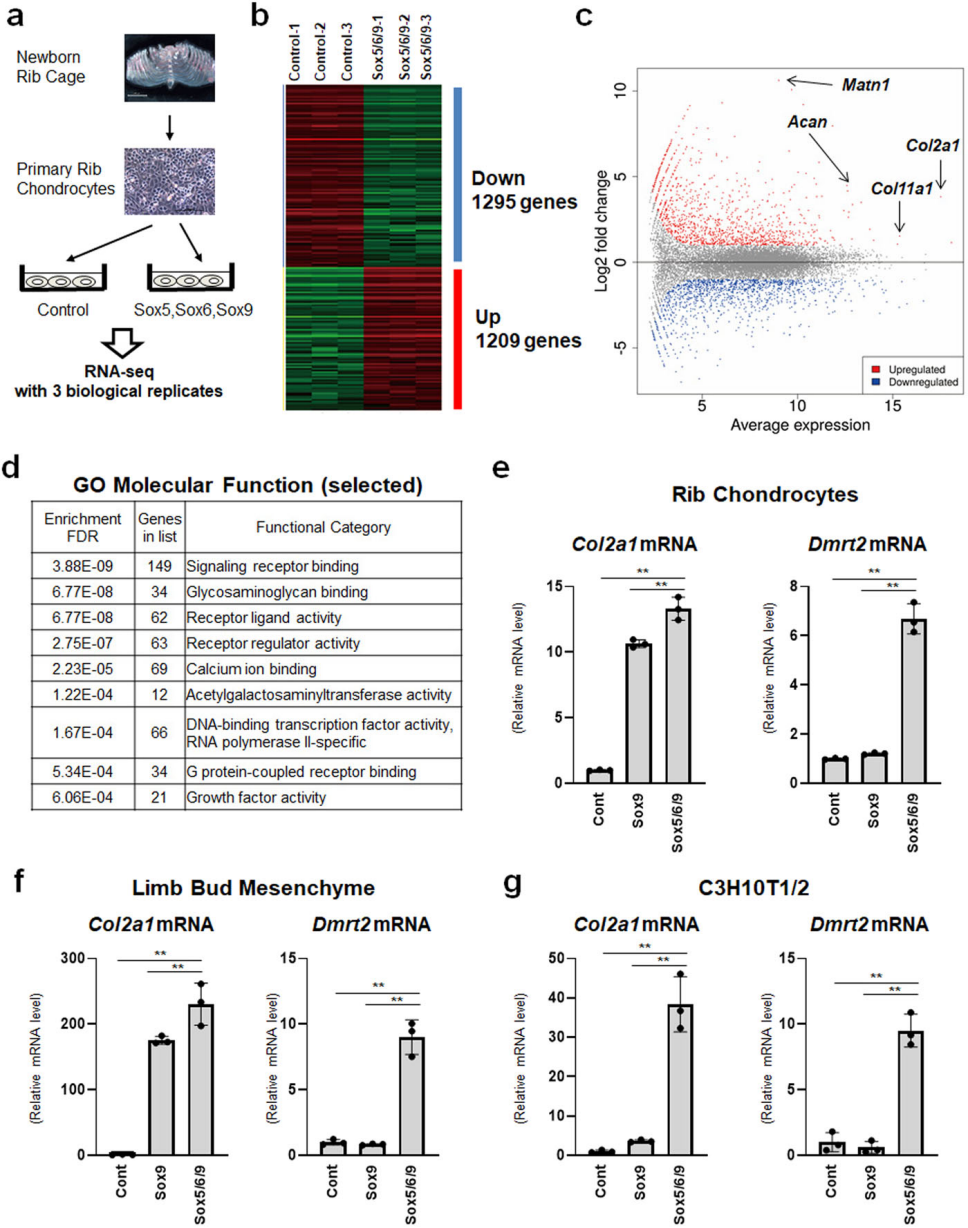
Identification of Dmrt2 as a downstream transcription factor of Sox5/6/9. **a** Schematic model of RNA-seq analysis using P0 newborn mouse rib chondrocytes with adenovirus-mediated overexpression of Sox5, Sox6, and Sox9. **b** Expression patterns of differentially expressed genes (DEGs). **c** MA plot analysis of DEGs. **d** Classification analysis of upregulated genes with GO molecular function. Selected molecular functions from Supplemental Data 1 are shown. **e**–**g** Total RNA was isolated from primary chondrocytes (**e**), limb bud mesenchyme from E12.5 mouse limb buds (**f**), and C3H10T1/2 cells (**g**) infected with control (Cont), Sox9 (Sox9), or Sox5 + Sox6 + Sox9 (Sox5/6/9) adenoviruses. *Col2a1* and *Dmrt2* mRNA expression levels were analyzed by RT-qPCR. The RNA level is indicated as the fold increase compared with that of the control. Data are shown as the mean ± s.d. (*n* = 3). **p < 0.01; one-way ANOVA followed by Tukey’s multiple comparison test.

To uncover the transcription factors involved in chondrocyte differentiation, we attempted to identify genes induced by Sox9 and its transcriptional cofactors, Sox5 and Sox6 (Sox5/6/9), in primary chondrocytes by RNA-seq analysis (Fig. 1a). RNA-seq analysis with three biological replicates identified 1295 downregulated and 1209 upregulated genes using thresholds of false discovery rate (FDR) < 0.05 and fold change > 2 (Fig. 1b). Relative to the levels in the control, primary chondrocytes overexpressing Sox5/6/9 exhibited the upregulation of many known Sox9 target genes, including *Col2a1* (14.3-fold), *Col11a1* (2.9-fold), *Acan* (22.3-fold), and *Matn1* (1706.2-fold) (Fig. 1c). Sox5/6/9 also promoted the expression of hypertrophic chondrocyte genes including *Ihh* (2.1-fold)*, Col10a1* (28.7-fold), and *Mmp13* (2.4-fold) (Supplementary Fig. 1). Enrichment analysis of GO molecular function revealed 66 DNA-binding transcription factors upregulated by Sox5/6/9 (Fig. 1d and Supplementary Table 1). Among these Sox5/6/9 target transcription factors, we were intrigued by the transcription factor Dmrt2. RNA-seq analysis showed the 5.5-fold increase of Dmrt2 in Sox5/6/9 overexpressing primary chondrocytes. Although severe skeletal defects associated with *DMRT2* mutations have been reported in humans and mice^20–22^, little is known about the functional roles of Dmrt2 in endochondral ossification. RT-qPCR confirmed that Sox5/6/9 upregulated *Col2a1* and *Dmrt2* expression in newborn rib chondrocytes, limb bud mesenchyme, and C3H10T1/2 cells (Fig. 1e–g). Consistent with the upregulation of *Dmrt2*, Sox5/6/9 also increased *Ihh* expression in C3H10T1/2 cells (Supplementary Fig. 2). In addition, among the seven *Dmrt* family transcription factors, only *Dmrt2* showed strong expression in the rib cartilage of newborn mice (Supplementary Fig. 3a), as well as Sox5/6/9-dependent induction in differentiating C3H10T1/2 cells (Supplementary Fig. 3b). Collectively, these findings suggest that the collaboration of Sox5, Sox6, and Sox9 induces the expression of *Dmrt2* in chondrocytes.

### Dmrt2 is expressed in chondrocytes during endochondral bone formation in vivo

**Fig. 2:**
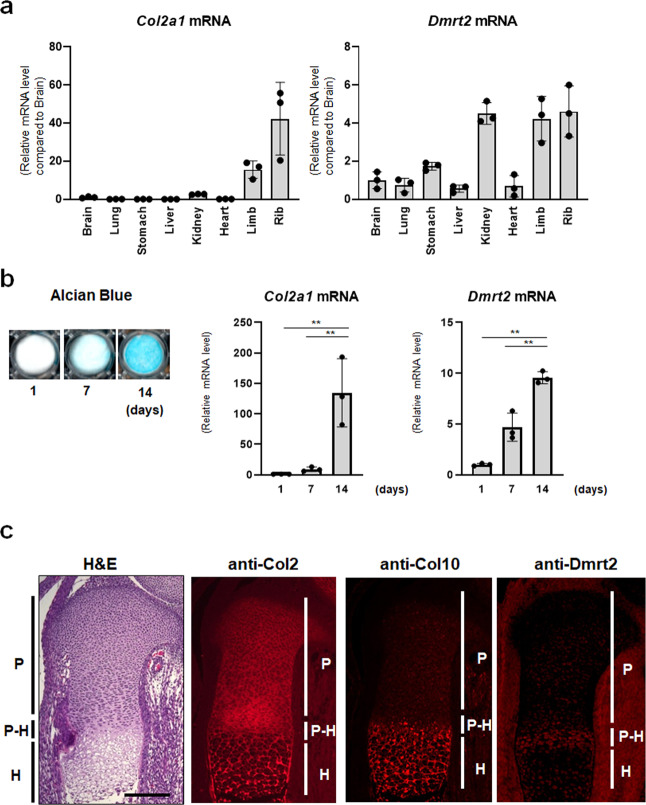
Expression of Dmrt2 in chondrocytes. **a** Tissue distribution of *Dmrt2* and *Col2a1* mRNAs in P0 newborn mouse tissues. Total RNA was isolated from the indicated tissues of P0 newborn mice and analyzed by RT-qPCR. The relative mRNA expression compared with that in brain is indicated, with the expression level in brain set as 1. Data are shown as the mean ± s.d. (*n* = 3). **b** ATDC5 cells were cultured in the presence of insulin*–*transferrin–selenium (ITS) for the indicated time and then stained with Alcian blue (left panel). Total RNA isolated from these cells was analyzed by RT-qPCR for *Col2a1* (middle panel) and *Dmrt2* expression (right panel). The RNA level is indicated as the fold increase compared with that on day 1. Data are shown as the mean ± s.d. (*n* = 3). **p < 0.01 (vs. day 1); one-way ANOVA followed by Tukey’s multiple comparison test. **c** Immunohistochemical analysis of Dmrt2 in growth plate chondrocytes. Sections of an E15.5 mouse tibia were subjected to H&E staining and immunocytochemical analyses using anti-Col2, -Col10, and -Dmrt2 antibodies. Scale bar: 200 μm. P proliferating chondrocytes, P-H pre-hypertrophic chondrocytes, H hypertrophic chondrocytes.

To examine whether *Dmrt2* is expressed in chondrocytes during endochondral bone formation, we first determined the tissue distribution of Dmrt2 by performing RT-qPCR. We observed relatively high expression of *Dmrt2*, as well as the Sox9 target gene *Col2a1*, in rib cartilage (Fig. 2a). In addition, *Dmrt2* expression increased during in vitro chondrocyte differentiation of ATDC5 cells in the presence of insulin–transferrin*–*selenium (ITS), as revealed by Alcian blue staining and *Col2a1* expression (Fig. 2b). Furthermore, immunofluorescence analysis of E15.5 mouse tibia sections revealed that Dmrt2 was highly expressed in pre-hypertrophic chondrocytes, but weakly expressed in proliferating or hypertrophic chondrocytes (Fig. 2c). Dmrt2 was also expressed in pre-hypertrophic chondrocytes around the secondary ossification center in 2-week-old mouse tibia, but its expression level was low in the growth plate of 4-week-old mouse tibia (Supplementary Fig. 4). The immunohistochemical analysis further demonstrated the colocalization of Sox9 and Dmrt2 in growth plate chondrocytes (Supplementary Fig. 5). Taken together, these findings suggest the possibility that Dmrt2 plays stage-specific roles in pre-hypertrophic chondrocytes during endochondral bone formation.

### Epigenetic regulation of Dmrt2 in chondrocytes

**Fig. 3:**
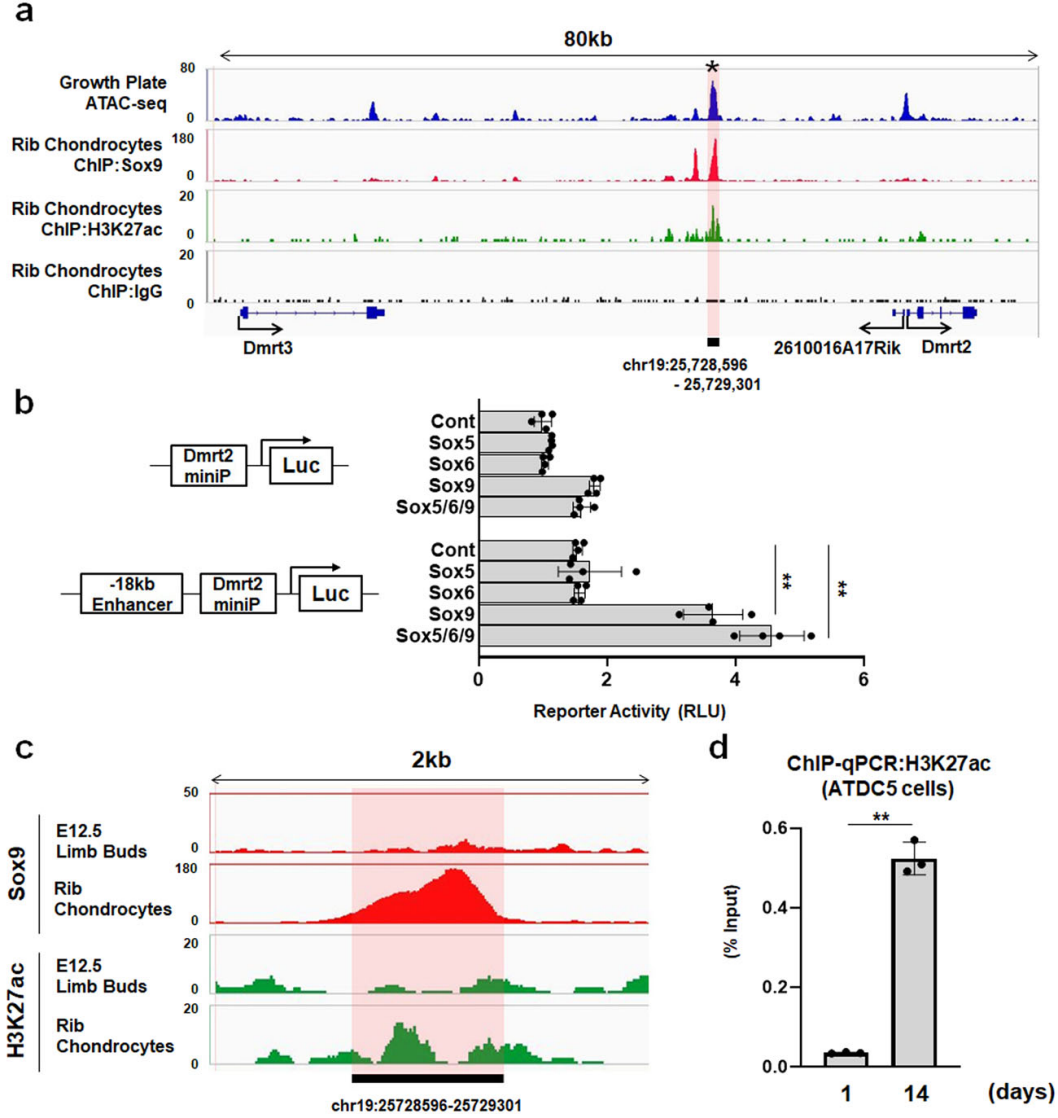
Epigenetic regulation of *Dmrt2* gene expression in chondrocytes. **a** ATAC-seq profiles of growth plate chondrocytes (GSE100585) and ChIP-seq profiles of primary newborn rib chondrocytes for Sox9, H3K27ac, and IgG (GSE69109) at *Dmrt2* gene loci. Note that strong ATAC-seq peaks overlapped with ChIP-seq peaks for Sox9 and H3K27ac. **b** HEK293 cells were transfected with reporter plasmids together with or without Sox9-bound enhancer and Sox5/6/9 as indicated. Luciferase activities were measured at 48 h after transfection. Data are shown as the mean ± s.d. (*n* = 4). **p < 0.01 (vs. Control); one-way ANOVA followed by Tukey’s multiple comparison test. **c** ChIP-seq profiles for Sox9 (GSE73225) and H3K27ac (GSE45456) in E12.5 limb buds and newborn primary rib chondrocytes (GSE69109). **d** ChIP-qPCR analysis for Sox9-bound enhancer of the *Dmrt2* gene using the anti-H3K27ac antibody in ATDC5 cells. Data are shown as the mean ± s.d. (*n* = 3). **p < 0.01 (vs. day 1); unpaired Student’s *t* test.

To investigate the molecular mechanism by which Sox5/6/9 regulates *Dmrt2* gene expression, we analyzed an epigenetic dataset of chondrocytes including ATAC-seq, which allows the genome-wide profiling of the open chromatin region^23^, and a ChIP-seq dataset^24^. We performed combination analysis of ATAC-seq profiles of growth plate chondrocytes (GSE100585) and ChIP-seq profiles of rib chondrocytes for Sox9, H3K27ac, and IgG (GSE69108). As shown in Fig. 3a, we found a strong peak of the open chromatin region (chr19:25,728,596–25,729,301) located 18 kb upstream of *Dmrt2* TSS (Fig. 3a). It should be noted that this region extensively overlaps with the peak of ChIP-seq for Sox9 and H3K27ac, an active enhancer mark of transcription^25^ (Fig. 3a). Consistent with the bioinformatic analyses, reporter assay indicated that Sox9 and Sox5/6/9 significantly upregulated the transcriptional activity on the enhancer region (Fig. 3b). These findings suggest that Sox9 upregulates Dmrt2 expression through an 18 kb upstream Sox9-bound enhancer.

We next examined whether the chromatin status of this Sox9-bound enhancer changes during chondrocyte differentiation. To achieve this, we obtained ChIP-seq datasets of E12.5 limb buds for Sox9 (GSE73225)^26^ and H3K27ac (GSE45456)^27^, and compared them with that of mature chondrocytes isolated from newborn ribs^24^. We found that Sox9 occupancy and active enhancer mark (H3K27ac) were very weak in E12.5 limb buds compared with the levels in rib chondrocytes (Fig. 3c). The chIP-qPCR analysis further demonstrated that the enrichment of H3K27ac in differentiated ATDC5 cells became significantly higher than that of undifferentiated ATDC5 cells (Fig. 3d). Taken together, these findings suggest that Sox9 directly regulates Dmrt2 expression through epigenetic regulation of the active enhancer.

### Dmrt2 is critical for endochondral bone formation in vivo

**Fig. 4:**
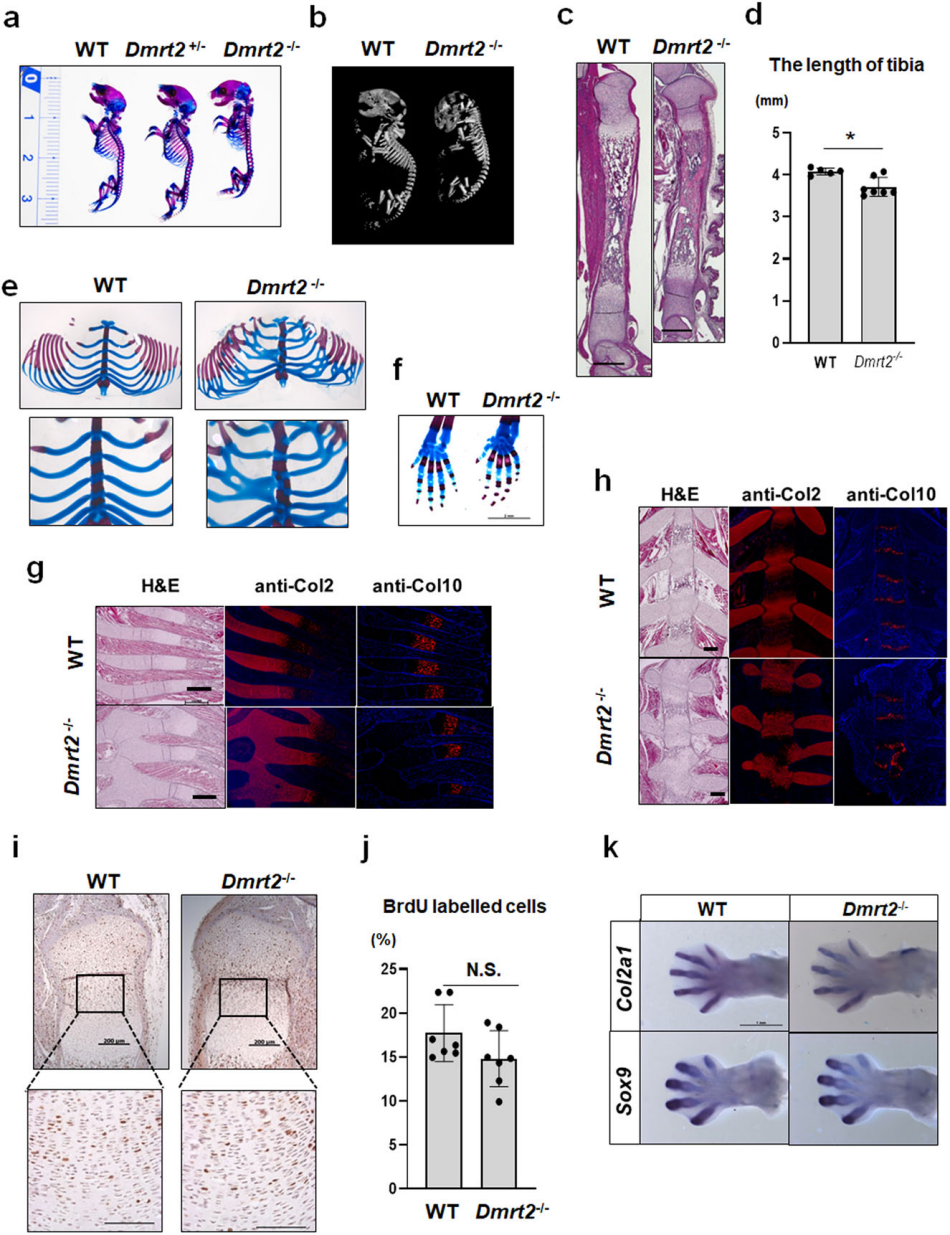
Dwarfism phenotype in *Dmrt2*^−/−^ mice. **a** Image of Alcian blue/Alizarin red S-stained skeletal preparations of P0 newborn WT, *Dmrt2*^*+/−*^, and *Dmrt2*^*−/−*^ littermate mice. **b** MicroCT images of P0 newborn WT and *Dmrt2*^*−/−*^ littermate mice. **c** Sections of tibiae from P0 newborn WT and *Dmrt2*^*−/−*^ littermate mice were examined by hematoxylin and eosin (H&E) staining. Scale bar: 500 μm. **d** Quantitative analysis of tibia lengths in P0 newborn WT and *Dmrt2*^*−/−*^ mice. Data are shown as the mean ± s.d. (WT, *n* = 5; *Dmrt2*^*−/−*^, *n* = 7). *p = 0.0061 (vs. WT); unpaired Student’s *t* test. **e**, **f** Photographs of Alcian blue/Alizarin red S-stained skeletal preparations of sternum, ribcage, and the hind paw of P0 newborn WT and *Dmrt2*^*−/−*^ littermate mice. Higher magnification of the sternum is shown in the lower panels of (**e**). **g**, **h** Sections of the ribcage (**g**) and sternum (**h**) from P0 newborn WT and *Dmrt2*^*−/−*^ littermate mice were examined by H&E staining and immunohistochemical analysis using antibodies against Col2 and Col10. Scale bars: 500 μm (**g**) and 200 μm (**h**). **i**, **j** BrdU labeling assay of WT and *Dmrt2*^*−/−*^ littermate mice. **i** Representative images of BrdU staining. Scale bars, 200 μm (upper panel) and 100 μm (lower panel). **j** Quantitative analysis of the proliferation rate of columnar chondrocytes. The ratio of BrdU-positive nuclei to total hematoxylin-positive nuclei was calculated. BrdU-positive nuclei in round resting and hypertrophic chondrocytes were excluded from the analysis. Data are shown as the mean ± s.d. (*n* = 7). N.S. not significant. **k** Whole-mount in situ analysis of *Col2a1* and *Sox9* in hindlimbs from E13.5 WT and *Dmrt2*^*−/−*^ littermate embryos. Scale bar: 1 mm.

We next tested whether Dmrt2 regulates endochondral bone formation in vivo by examining *Dmrt2*-deficient (*Dmrt2*^−/−^) mice. *Dmrt2*^−/−^ mice died soon after birth, as reported previously^20^. Newborn *Dmrt2*^*−/−*^ mice showed a dwarf phenotype in contrast to wild-type (WT) and *Dmrt2* heterozygous mice, as determined by skeletal preparations and microcomputed tomography (microCT) analysis (Fig. 4a, b). In addition, *Dmrt2*^*−/−*^ mice exhibited significantly shorter tibiae than WT mice (Fig. 4c, d) and severely disrupted rib development, characterized by disorganized rib fusion and abnormal ossification in the sternum (Fig. 4e). In contrast, abnormal skeletal patterning was not observed in the hind paws of *Dmrt2*^*−/−*^ mice (Fig. 4f ).

To investigate the potential role of Drmt2 in chondrocyte differentiation in vivo, we evaluated the organization of growth plate chondrocytes in newborn mice. Immunofluorescence analysis revealed that Col2-positive rib cartilage of *Dmrt2*^*−/−*^ mice showed truncation, fusion, and bifurcation, and the Col10-positive hypertrophic chondrocyte zone in *Dmrt2*^*−/−*^ mice was shortened relative to that in WT mice (Fig. 4g). We also found that Col2-positive and Col10-positive chondrocytes were disorganized in the sternum of *Dmrt2*^*−/−*^ mice relative to the pattern in WT mice (Fig. 4h).

To determine whether impaired endochondral bone formation might arise from suppressed chondrocyte proliferation in *Dmrt2*^*−/−*^ mice, we performed BrdU labeling assays of embryos. We found that the chondrocyte proliferation rate was not significantly reduced in *Dmrt2*^*−/−*^ mice relative to the level in WT mice (Fig. 4i, j). Whole-mount in situ hybridization of WT and *Dmrt2*^*−/−*^ E13.5 embryos did not show significant differences in *Col2a1-* and *Sox9*-positive chondrocytes (Fig. 4k), consistent with our findings that *Drmt2* is not yet expressed at this stage. These findings suggest that aberrant chondrocyte differentiation at a later stage, not proliferation, likely contributes to the skeletal abnormalities in *Dmrt2*^*−/−*^ mice.

**Fig. 5:**
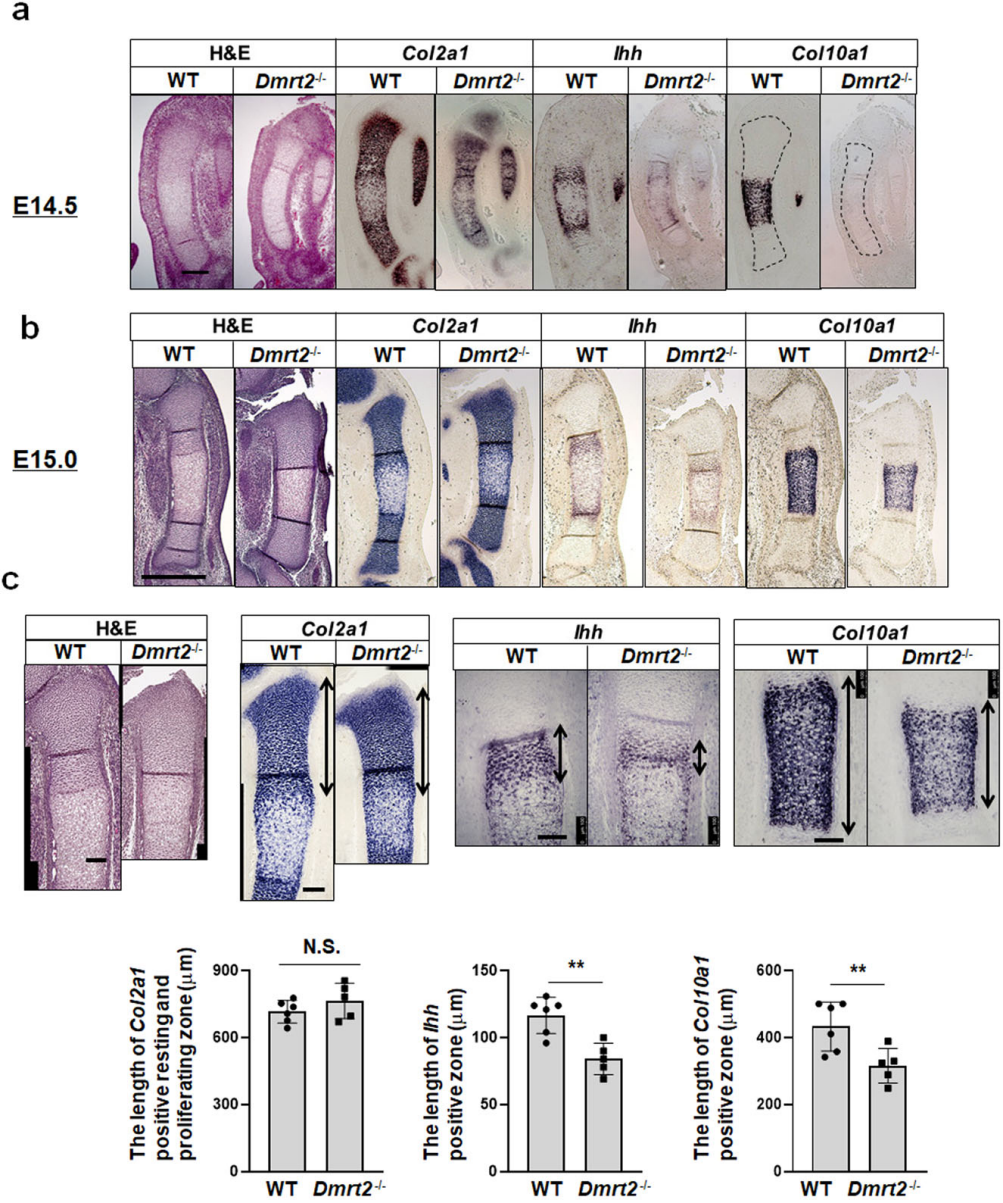
Delayed onset of chondrocyte hypertrophy in *Dmrt2*^−/−^ mice. **a**, **b** Sections of tibiae from E14.5 (**a**) and E15.0 (**b**) WT and *Dmrt2*^*−/−*^ littermate embryos were examined by hematoxylin and eosin (H&E) staining and RNA in situ hybridization analysis using antisense probes against *Col2a1*, *Ihh*, and *Col10a1*. Scale bars: 200 μm (E14.5), 500 μm (E15.0). **c** Quantitative analysis of *Col2a1*-positive resting and proliferating length, *Ihh*-positive length, and *Col10a1*-positive length in E15.0 WT and *Dmrt2*^*−/−*^ mice. Double-headed arrows indicate the measured length in the representative images. Data are shown as the mean ± s.d. (WT, *n* = 6; *Dmrt2*^*−/−*^, *n* = 5). *p = 0.0023 (*Ihh*-positive length), *p* = 0.0149 (*Col10a1*-positive length) (vs. WT); unpaired Student’s *t* test.

We further examined how the loss of *Dmrt2* in mice affects the chondrocyte differentiation program during endochondral bone formation. At E14.5, WT embryos normally exhibited *Ihh-* and *Col10a1*-positive chondrocytes in the tibial growth plate, but *Dmrt2*^*−/−*^ embryos showed weak *Ihh* expression and lacked *Col10a1*-positive chondrocytes (Fig. 5a). At E15.0, the *Col2a1*-positive chondrocyte zone in WT embryos was completely divided by the *Ihh-* and *Col10a1-*positive chondrocytes (Fig. 5b), whereas the boundary between Col2a1-positive and Col10a1-positive chondrocyte zone was unclear in Dmrt2^−/−^ embryos (Fig. 5b). Next, we quantitatively assessed in situ hybridization sections to measure the lengths of the *Col2a1*-expressing zone (resting and proliferating chondrocyte zone), *Ihh*-expressing zone (pre-hypertrophic chondrocyte zone), and *Col10a1*-expressing zone (hypertrophic zone) in the tibiae of E15.0 mice (Fig. 5c). We did not observe significant differences in the lengths of the resting/proliferating zone between WT and *Dmrt2*^*−/−*^ embryos (Fig. 5c). However, the *Ihh*-positive pre-hypertrophic and *Col10a1*-positive hypertrophic zones were significantly shorter in *Dmrt2*^*−/−*^ embryos than in WT embryos (Fig. 5c). In situ hybridization analysis of rib cartilage also demonstrated the shortening of the *Ihh-* and *Col10a1*-positive zone of newborn *Dmrt2*^*−/−*^ mice compared with that of WT mice (Supplementary Fig. 6). These findings suggest that impaired endochondral bone formation is partly responsible for the skeletal abnormalities in *Dmrt2*^*−/−*^ mice.

### Dmrt2 promotes late chondrogenesis by functional interaction with Runx2

**Fig. 6:**
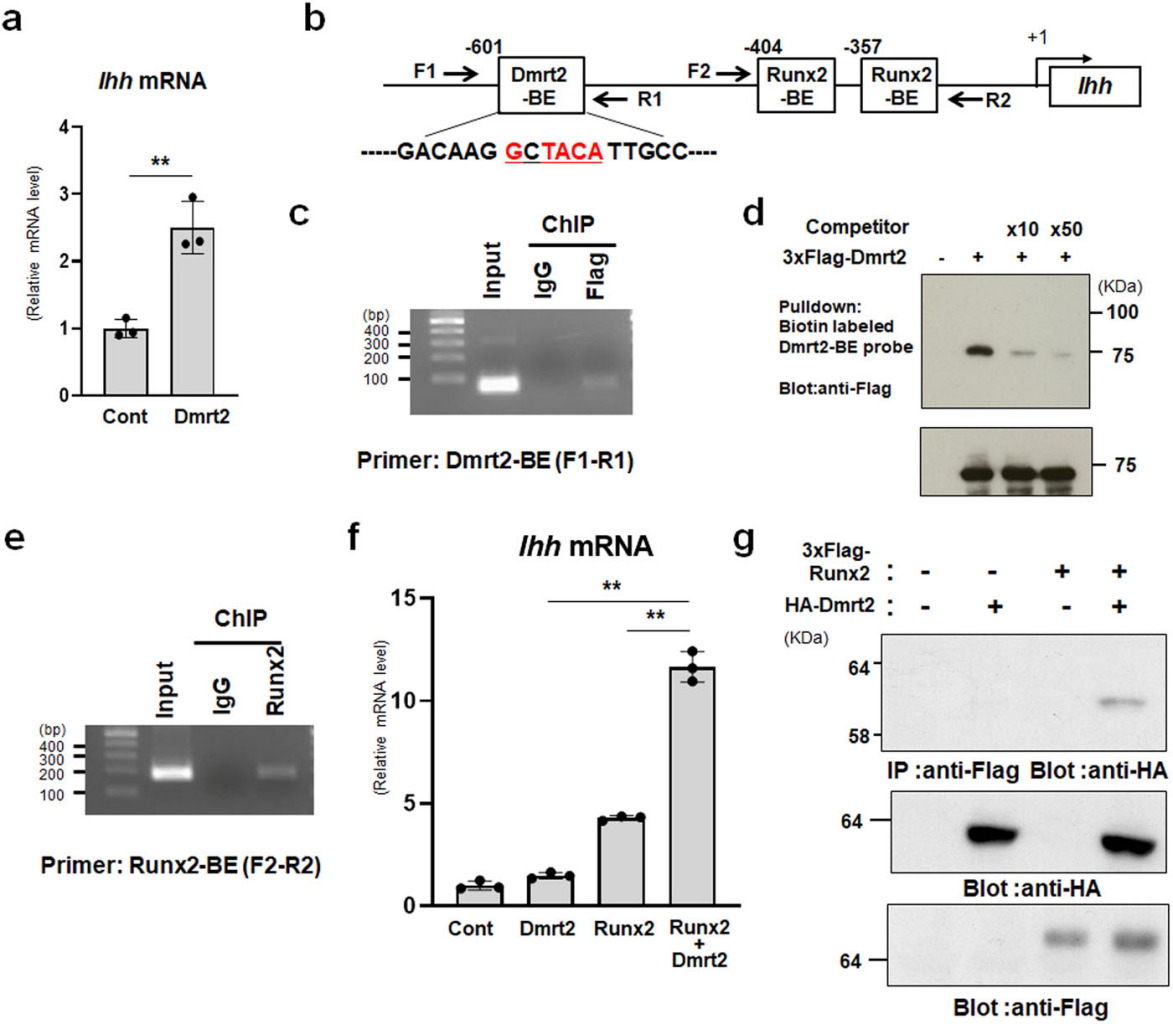
Dmrt2 promotes Ihh expression in collaboration with Runx2. **a** Primary chondrocytes were infected with the control (Cont) or Flag-tagged-Dmrt2 adenovirus, and *Ihh* expression was analyzed by RT-qPCR. Data are shown as fold changes normalized to Cont (mean ± s.d.) (*n* = 3). **p < 0.01 (vs. WT); Student’s *t* test. **b** Schematic representation of the putative Dmrt2-binding element (-BE) (GnTACA) in the mouse *Ihh* gene promoter. **c** ChIP assay using normal IgG and anti-Flag antibody. The binding of Flag-Dmrt2 to the *Ihh* gene promoter in primary chondrocytes was examined by PCR. **d** DNA pull-down assays using the Dmrt2-binding element in the mouse *Ihh* gene promoter. Samples precipitated with a biotin-labeled Dmrt2-BE oligonucleotide (upper panel) and cell lysates (lower panel) were examined by immunoblotting with an anti-Flag antibody. **e** ChIP assays using normal IgG and anti-Runx2 antibodies. The binding of Runx2 to the *Ihh* gene promoter in primary chondrocytes was examined by PCR. **f** Dmrt2 and Runx2 synergize to induce *Ihh* mRNA expression in primary chondrocytes. The RNA level is indicated as the fold increase compared with the level in the control. Data are shown as the mean ± s.d. (*n* = 3). **p < 0.01; one-way ANOVA followed by Tukey’s multiple comparison test. **g** Dmrt2 physically associates with Runx2. Cell lysates were immunoprecipitated with anti-Flag antibody and then immunoblotted with anti-HA antibody (top). Cell lysates were immunoblotted with anti-HA (middle) or anti-Flag (bottom) antibodies.

Given the delay in chondrocyte hypertrophy observed upon the loss of *Drmt2*, we determined whether Dmrt2 promotes the initiation of this process. Importantly, *Dmrt2* overexpression in primary chondrocytes significantly upregulated the expression of *Ihh* (Fig. 6a). We noticed that the promoter of mouse *Ihh*, a specific marker of pre-hypertrophic chondrocytes, contains a consensus *Dmrt2*-binding element (BE, GnTACA) (Fig. 6b). We found that Flag-tagged Dmrt2 bound to this element within the *Ihh* gene promoter in primary chondrocytes as determined by ChIP assay (Fig. 6c). A DNA-pulldown assay using a biotinylated Dmrt2-binding element also demonstrated that Dmrt2 directly bound to this element, and the Dmrt2 binding activity was decreased by a non-biotinylated probe in a dose-dependent manner (Fig. 6d). These findings collectively suggest that Dmrt2 directly promotes *Ihh* expression during endochondral bone formation.

The transcription factor Runx2 directly regulates *Ihh* expression, consequently stimulating chondrocyte hypertrophy^9^. We found that the Runx2 binding element in the *Ihh* proximal promoter is located close to the Dmrt2-binding element (Fig. 6b) and confirmed the direct binding of Runx2 to the *Ihh* gene promoter by ChIP assay (Fig. 6e). Thus, we hypothesized that Dmrt2 functionally collaborates with Runx2 to regulate *Ihh* expression. To prove this hypothesis, we ectopically expressed Dmrt2 and Runx2 in primary chondrocytes and examined *Ihh* expression. We confirmed that overexpression of Dmrt2 had no effect on Runx2 expression by western blotting (Supplementary Fig. 7). Notably, primary chondrocytes overexpressing both Dmrt2 and Runx2 displayed higher *Ihh* mRNA levels than cells overexpressing either Drmt2 or Runx2 alone, suggesting a synergistic interaction (Fig. 6f). Dmrt2 and Runx2 also synergized to induce the expression of other Runx2 targets in primary chondrocytes, including *Alpl* (alkaline phosphatase), *Col10a1*, and *Tcf7* (Supplementary Fig. 8). Furthermore, coimmunoprecipitation experiments indicated a physical association of Dmrt2 with Runx2 (Fig. 6g). Taken together, these findings suggest that Dmrt2 interacts with and enhances Runx2 function at the target gene, *Ihh*.

### Impaired Runx2 function in *Dmrt2*^−/−^ chondrocytes

**Fig. 7:**
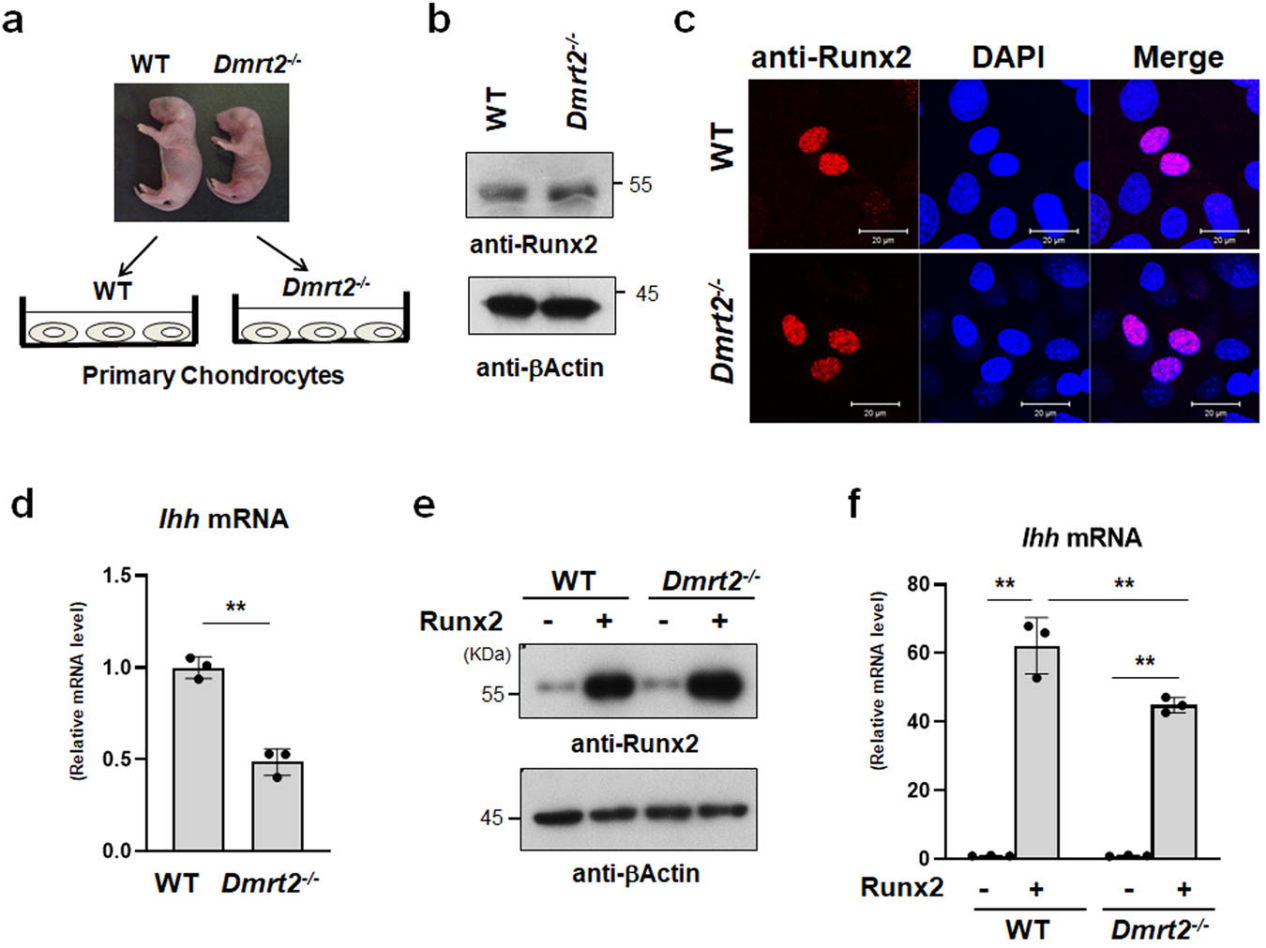
Impaired Runx2 function in *Dmrt2*^−/−^ chondrocytes. **a** The method to examine the effects of Runx2 in WT and *Dmrt2*^*−/−*^ chondrocytes. **b** Primary chondrocytes were isolated from P0 newborn WT and *Dmrt2*^*−/−*^ littermate mice and cultured for 3 days. Cell lysates were analyzed by immunoblotting with anti-Runx2 and anti-β-actin antibodies. **c** Nuclear localization of Runx2 in primary chondrocytes of WT and *Dmrt2*^*−/−*^ chondrocytes. Confocal sections were visualized under a fluorescence microscope. Scale bar: 20 μm. **d** Total RNA was isolated from primary chondrocytes of WT and *Dmrt2*^*−/−*^ littermate mice, and then *Ihh* mRNA expression was determined by RT-qPCR. Data are shown as fold changes normalized to WT (mean ± s.d., *n* = 3). **p < 0.01 (vs. WT); unpaired Student’s *t* test. **e** Primary chondrocytes from WT and *Dmrt2*^*−/−*^ littermate mice were infected with Runx2 adenovirus and cultured for 4 days. Cell lysates were analyzed using immunoblotting with anti-Runx2 and anti-β-actin antibodies. **f** Primary chondrocytes from WT and *Dmrt2*^*−/−*^ littermate mice were infected with the control (−) or Runx2 adenovirus and then cultured for 4 days. Total RNA was isolated and *Ihh* mRNA expression was determined by RT-qPCR. The RNA level is indicated as the fold increase compared with that of the WT control. **p < 0.01; two-way ANOVA followed by Tukey’s multiple comparison test.

Finally, we examined whether the activities of Runx2 are affected in *Dmrt2*^*−/−*^ mice. To this end, we isolated primary chondrocytes from newborn WT and *Dmrt2*^*−/−*^ mice and examined the effect of Dmrt2 on Runx2 function by monitoring *Ihh* expression (Fig. 7a).

WT and *Dmrt2*^*−/−*^ chondrocytes showed similar levels of endogenous Runx2 protein (Fig. 7b) and we also found that loss of Dmrt2 did not affect the nuclear localization of endogenous Runx2 in primary chondrocytes, which suggests that Drmt2 does not affect the expression and stability of Runx2 (Fig. 7c). Consistent with the *ISH* analyses (Fig. 5a, b), we found significantly reduced expression of *Ihh*, the Runx2 target gene, in *Dmrt2*^*−/−*^ chondrocytes compared with the level in WT chondrocytes (Fig. 7d). When we introduced Runx2 at equal levels into primary chondrocytes isolated from WT and *Dmrt2*^*−/−*^ mice (Fig. 7e), overexpression of Runx2 induced *Ihh* expression in WT chondrocytes, but this effect was reduced in *Dmrt2*^*−/−*^ chondrocytes (Fig. 7f). These findings support the importance of the interaction of Dmrt2 with Runx2 for *Ihh* expression.

## Discussion

Endochondral bone formation is achieved through successive chondrocyte differentiation steps that are strictly regulated in a spatiotemporal manner by various transcription factors. Previous biochemical and mouse genetic studies have shown that Sox9 regulates multiple steps of chondrocyte differentiation including early chondrogenesis and chondrocyte hypertrophy^15,16^. Although the mechanisms by which Sox9 and its target genes regulate early chondrogenesis have been well studied, Sox9 target genes and their functional roles in chondrocyte hypertrophy remain poorly understood. In the present study, we discovered that the transcription factor Dmrt2 is selectively expressed in pre-hypertrophic chondrocytes and is induced by Sox5/6/9. We also found that Dmrt2 contributes to the endochondral bone formation by promoting Runx2 functions (Supplementary Fig. 9). Our results suggest that Dmrt2 coordinates successive chondrocyte differentiation processes during skeletal development.

Runx2 is critical for chondrocyte hypertrophy by directly regulating *Ihh*^9^. We uncovered the novel mechanism by which Runx2 induces *Ihh* expression through functional collaboration with Dmrt2. It should be noted that both *Runx2*^*−/−*^ mice and *Dmrt2*^*−/−*^ mice die shortly after birth due to respiratory failure, suggesting the functional similarity of Dmrt2 and Runx2^20,28^. Although Runx2 expression is detectable from pre-hypertrophic to hypertrophic chondrocytes^29^, Dmrt2 is highly expressed in pre-hypertrophic chondrocytes but weakly expressed in hypertrophic chondrocytes (Fig. 2c). These findings suggest that the synergistic function of Runx2 and Dmrt2 mainly occurs in pre-hypertrophic chondrocytes and that Runx2-dependent *Ihh* expression decreases in hypertrophic chondrocytes. However, the mechanisms of selective *Ihh* expression in pre-hypertrophic chondrocytes have remained unclear. We speculate that biological interaction between Runx2 and Dmrt2, at least in part, accounts for the selective expression of *Ihh* in growth plate chondrocytes.

In addition to Ihh, the overexpression of Dmrt2 synergistically induced the expression of Runx2 target genes including *Col10a1* (Supplementary Fig. 8), whereas Dmrt2-deficient mice showed a shortened *Col10a1*-positive zone (Fig. 5). These findings led us to hypothesize that Dmrt2 directly regulates chondrocyte hypertrophy. However, there are several limitations that impede our ability to prove this hypothesis. First, primary chondrocytes mainly include resting, proliferating, and pre-hypertrophic chondrocytes, so it is impossible to correctly evaluate the phenotype of hypertrophic chondrocytes in vitro. Second, the generation and characterization of hypertrophic chondrocyte-specific Dmrt2 KO are impossible because Dmrt2 floxed mice are not available. Further studies are necessary to investigate the direct effects of Dmrt2 on chondrocyte hypertrophy.

We show that Dmrt2 is important for *Ihh* expression, but the dwarf phenotype of Dmrt2 mice was not severe compared with that of *Ihh*-deficient mice^30^ and *Runx2*-deficient mice^9^. These findings imply that other transcription factors compensate for the loss of Dmrt2. Previous studies reported several transcription factors, including C/EBPβ, Atf6a, Gli2, Maml1, Sp7, and Zfp521, that work as transcriptional activators of Runx2 in chondrocytes to promote chondrocyte hypertrophy^31–36^. Among these, C/EBPβ and Atf6a synergistically induce *Ihh* expression in collaboration with Runx2^33,35^. We speculate that C/EBPβ and Atf6a partially compensate for the loss of *Ihh* expression in Dmrt2^*−/−*^ mice.

We identified Dmrt2 as a transcription factor functioning downstream of Sox5/6/9 in primary chondrocytes. Several studies have reported the targets of Sox9 or Sox5/6/9 in chondrocytes, but these targets promote early chondrogenesis and negatively regulate chondrocyte hypertrophy^37,38^. Yamashita et al. reported that Bapx1, a direct target of Sox9, suppressed Runx2 activity in chondrocytes^38^. In addition, Saito et al.^37^ found that S100A1 and S100B1 are directly regulated by Sox5/6/9 and suppress chondrocyte hypertrophy and maturation. We also identified the transcription factor Sp6 as a Sox9 target and showed that its deletion in mice results in a dwarf phenotype and impaired limb development^39,40^. These results fit the idea that Sox9 plays essential role in chondrogenesis. However, it is now established that Sox9 is required for chondrocyte hypertrophy^15,16^ and our findings suggest that target genes of Sox5/6/9 positively regulate chondrocyte hypertrophy. In addition to Dmrt2, FoxA2 and AP-1 family members, which were identified as Sox5/6/9-inducible genes (Supplemental Data 1), promote chondrocyte hypertrophy through functional interaction with Sox9^16,41^. Thus, other Sox5/6/9 target genes and their biological interactions with hypertrophic transcription factors warrant further investigation.

We found that Sox9 directly bound to the enhancer region of the *Dmrt2* gene located 18 kb upstream of its TSS by epigenetic analysis using ATAC-seq and ChIP-seq datasets. This region showed both open chromatin and H3K27ac, a histone mark for active enhancers (Fig. 3a). Ohba et al. previously reported that Sox9 has two distinct modes of action in chondrocytes^24^. Class I elements exist around the TSS of highly expressed genes not specific to chondrogenesis and Class II elements represent active enhancers that promote chondrocyte gene expression through the direct binding of Sox9. Intriguingly, a Sox9-occupied region located 18 kb upstream of Dmrt2 TSS belongs to Class II Sox9 binding elements^24^, which suggests that Dmrt2 is the chondrocyte-specific Sox9 target gene in chondrocytes.

Although the ChIP-seq dataset and reporter assay demonstrated that Sox9 is responsible for the 18 kb upstream enhancer of the Dmrt2 gene, Sox9 alone failed to increase Dmrt2 expression in primary chondrocytes, limb bud mesenchyme, and C3H10T1/2 cells (Fig. 1e–g). We propose several possibilities to explain this. First, appropriate amounts of Sox5 and Sox6 are necessary to induce the transcription of Dmrt2. Previous studies have shown that Sox5 and Sox6 secure the DNA binding activity of Sox9 and cooperatively promote chondrocyte gene expression through super-enhancers at the genome-wide level^14,42^. Liu et al.^14^ also reported that Sox6 and Sox9 bind genomic regions in the vicinity of each other. It would be interesting to examine whether Sox6 binds a genomic region located close to the Sox9 binding region 18 kb upstream of Dmrt2 TSS. Second, an unknown epigenetic mechanism controlled by Sox5 and Sox6 is necessary for the induction of the Dmrt2 gene. Many histone-modifying enzymes including demethylases and acetyltransferases are necessary for activating transcription in chondrocytes. Previous biochemical and epigenetic studies showed that Sox9 functionally interacts with CBP/P300, which works as a histone acetyltransferase^24,43^. Our group has shown that the histone demethylase PHF2 associates with Sox9 to promote Sox9 target gene expression in chondrocytes^44^. Although we did not provide direct evidence showing that Sox5 and Sox6 control histone modification, it is likely that they upregulate Dmrt2 expression through epigenetic remodeling. More studies are necessary to uncover the precise molecular mechanism underlying the effects of Sox5 and Sox6 to activate Dmrt2 gene expression in chondrocytes.

Sox5/6/9 increase *Dmrt2* mRNA and are strongly expressed in resting and proliferating chondrocytes, and therefore we first predicted that Dmrt2 is widely expressed in resting and proliferating chondrocytes. Unexpectedly, immunohistochemical analysis revealed that Dmrt2 protein is selectively expressed in pre-hypertrophic chondrocytes (Fig. 2c). These findings raise the possibility that other unknown transcription factors that are selectively expressed in pre-hypertrophic chondrocytes are required to accelerate Dmrt2 expression. Alternatively, post-transcriptional regulation of Dmrt2 controls pre-hypertrophic-specific protein expression. This complexity has been found to be exhibited by many genes expressed in chondrocytes of the growth plate. For instance, Ihh expression is known to be limited to pre-hypertrophic chondrocytes, but Runx2, which directly regulates Ihh, is widely expressed in both pre-hypertrophic and hypertrophic chondrocytes^9,45^. In addition, PTHrP is only detectable in the periarticular region, even though the Gli family, signaling molecules of Ihh, are diffusely distributed in the round and proliferating chondrocytes^5,46^. The molecular mechanisms that control the specific expression of chondrocyte genes are not fully understood, so further studies are necessary to clarify them.

Dmrt2 contains a highly conserved DNA binding domain called the DM domain, but its sequence similarity with other Dmrt family members is low outside the DM domain^19^. The DM domain recognizes a consensus sequence and physically interacts with DNA in the minor groove^47^. Dmrt proteins are predicted to bind DNA as heterodimers or homodimers with other Dmrt family proteins^48^. Notably, Dmrt proteins act as bifunctional transcriptional regulators to activate or repress transcription^19^. For instance, Dmrt1 inhibits Stra8 but activates Sohlh1 in germ cells, which prevents meiosis and promotes spermatogonial development^49^. Whether Dmrt1 activates or represses transcription appears to depend on motifs around Dmrt1 binding sites, which suggests that the function of Dmrt1 depends on co-activators and co-repressors^50^. These findings raise the possibility that Dmrt2 also exerts a reciprocal function in chondrocytes in addition to the increase of Runx2 function. Notably, pre-hypertrophic chondrocytes are the transition stage from a proliferating to a hypertrophic state, at which the expression of proliferating chondrocyte-specific genes such as *Col2a1* and *Aggrecan* should be inhibited at the transcriptional level. It would be interesting to determine whether Dmrt2 controls chondrocyte differentiation by suppressing Sox9-dependent gene expression but promoting Runx2-dependent gene expression in pre-hypertrophic chondrocytes. The regulation of Sox9 function by Dmrt2 during endochondral bone formation awaits further investigation.

In conclusion, our work suggests a novel role for Dmrt2 during endochondral bone formation as a transcriptional coactivator of Runx2. These findings increase our understanding of the molecular mechanisms of endochondral bone formation and provide new insights into the transcription factor network controlling skeletal development.

## Methods

### Cell culture and reagents

The mouse fibroblast-like cell line C3H10T1/2 and mouse teratocarcinoma cell line ATDC5 were purchased from the RIKEN Cell Bank (Ibaraki, Japan). These cells were cultured at 37 °C in a humidified 5% CO_2_ incubator with Dulbecco’s modified Eagle’s medium (DMEM; Sigma-Aldrich, St. Louis, MO, USA) containing 10% fetal bovine serum (FBS) and a 1:1 mixture of DMEM and Ham’s F-12 medium (Sigma-Aldrich) containing 10% FBS. Insulin–transferrin–sodium selenite supplement (ITS; Roche, Basel, Switzerland) was used to induce chondrocyte differentiation in ATDC5 cells. Primary chondrocytes were isolated in accordance with a protocol described by Gartland et al.^51^. Briefly, rib cartilage was dissected from newborn mice and soft tissue was removed. Then, rib cartilage was digested with 0.1% collagenase D (Roche) and 0.5% trypsin (Life Technology, Carlsbad, CA, USA) for 6 h at 37 °C. Primary chondrocytes were collected by centrifugation and resuspended with DMEM containing 10% FBS and antibiotics. Cells within two passages were used for experiments as primary chondrocytes.

### Generation of adenoviruses

cDNAs of Flag-tagged mouse Dmrt2, HA-tagged mouse Sox9, mouse Sox5, mouse Sox6, and mouse Runx2 were amplified using Pfu DNA polymerase and subcloned into pAXCAwt vectors (TAKARA Bio, Shiga, Japan). Recombinant adenoviruses were generated using the COS-TPC method by transfection of a recombinant cosmid and the DNA-TPC adenovirus genome into 293 cells^44^. C3H10T1/2 cells, ATDC5 cells, and primary chondrocytes were infected with adenoviruses at a multiplicity of infection (MOI) of 20 unless indicated otherwise.

### RNA-seq

Total RNA was extracted from primary chondrocytes in which the control vector or Sox5/6/9 were adenovirally expressed using NucleoSpin RNA II (Macherey-Nagle, Duren, Germany). Total RNA library preparation was performed using a TruSeq Stranded mRNA sample prep kit (Illumina, San Diego, CA, USA), in accordance with the manufacturer’s protocol. Sequencing was performed on an Illumina HiSeq 2500 platform in 75-base single-end mode. Illumina Casava1.8.2 software was used for base-calling. Sequenced reads were mapped to the mouse reference genome sequence (mm10) using TopHat v2.0.13 in combination with Bowtie2 ver. 2.2.3 and SAMtools ver. 0.1.19.

RNA-seq data were analyzed using iDEP (integrated Differential Expression and Pathway analysis)^52^. Briefly, read count data of three replicates for control and Sox5/6/9 were generated and uploaded to the iDEP website (http://bioinformatics.sdstate.edu/idep/). Differentially expressed genes (DEGs) were identified using a threshold of FDR < 0.05 and minimal fold change > 2. The raw data have been deposited in the NCBI Gene Expression Omnibus database (GEO GSE155118).

### Reverse-transcription polymerase chain reaction (RT-PCR) and RT-qPCR

Total RNA was isolated using a Nucleo Spin RNA Plus kit. cDNA was synthesized using ReverTra Ace^®^ qPCR RT Master Mix (TOYOBO, Osaka, Japan). For RT-PCR analysis, cDNA was amplified using KOD FX (TOYOBO) and then the PCR products were electrophoresed in a 1.6% agarose gel with ethidium bromide. Primer pairs used for RT-PCR analysis are listed in Supplemental Table S2. For RT-qPCR analysis, cDNA was amplified with EagleTaq Universal Master Mix (ROX) using a StepOnePlus Real-Time PCR System (Applied Biosystems, Foster City, CA, USA). Primers and TaqMan probes used for cDNA amplification are listed in Supplementary Table S2. The mRNA expression was normalized to β-actin expression levels. Uncropped images of RT-PCR are included in Supplementary Fig. 10.

### Epigenetic datasets and analysis

ChIP-seq and ATAC-seq datasets were downloaded from the GEO database. We obtained GSE69109 ChIP-seq profiles of newborn mouse rib chondrocytes for Sox9 and H3K27ac^24^. ChIP-seq profiles of E12.5 limb buds for Sox9 were obtained from GSE73225^27^ and H3K27ac from GSE45456^26^. ATAC-seq datasets of growth plate chondrocytes were downloaded from GSE100585^23^.

FASTQ data of ChIP-seq and ATAC-seq were aligned to the mouse genome (mm10) using Bowtie and peak calling was performed using MACS software with the default settings (*p* value cut-off = 1.00e − 05). ChIP-seq and ATAC-seq data were visualized using the Integrative Genomics Viewer.

### Reporter assay

*Dmrt2* minimal promoter (−100 to +10) and Sox9 binding region located 18 kb upstream of the *Dmrt2* TSS were introduced upstream of the luciferase gene. Reporter genes were co-transfected with the expression vectors and *Renilla* into HEK293 cells using the FuGENE6 reagent. After 48 h of transfection, the cells were lysed and luciferase activity was measured using specific substrates in a luminometer (Promega, Fitchburg, WI, USA), in accordance with the manufacturer’s protocol. Luciferase activity was normalized by *Renilla*.

### Mice

*Dmrt2*^*−/−*^ mice were originally generated by Dr. Randy L. Johnson (Department of Biochemistry and Molecular Biology, The University of Texas MD Anderson Cancer Center)^20^. They were maintained and kindly provided by Dr. David Zarkower (Developmental Biology Center and Department of Genetics, Cell Biology, and Development, University of Minnesota) for the experiments. To determine the genotypes of the mice, genomic DNA was purified from mouse tails and examined by PCR using KOD FX Neo (Toyobo). Primer pairs for genotyping were as follows: WT, sense 5′-CTGGACCCGAGTACAGTTCC-3′, and antisense 5′-AATGGTGCGTTCAACTCAGG-3′; KO, sense 5′-TGCGGAGGGCTGGATCTTAAGGAG-3′ and antisense 5′-AGGGGGTGGGGATTTGACACCATC-3′. The PCR product of WT mice was 830 bp and that of KO mice was 270 bp.

All mice were maintained on the C57BL/6 background. Littermate embryos were used for histological analysis. All animal experiments were performed using protocols approved by the Animal Committee of Osaka University Graduate School of Dentistry.

### Skeletal preparation

The skin of the mice was removed and fixed in 95% ethanol overnight. Cartilage tissues were stained with 1.5% Alcian blue followed by staining of bone tissues with 0.02% Alizarin red S. Skeletal samples were photographed under a stereoscopic microscope.

### Immunohistochemistry

Samples were fixed with 4% buffered paraformaldehyde, embedded in paraffin, and cut into 4-μm-thick sections. Paraffin-embedded sections were deparaffinized and rehydrated, followed by hematoxylin and eosin (H&E) staining. For immunohistochemical analysis, antigen retrieval was performed by incubation in DAKO REAL target retrieval solution for 10 min at 90 °C, followed by blocking with 1% bovine serum albumin in phosphate-buffered saline (PBS). Immunohistochemistry was performed using the following antibodies: anti-Dmrt2 (#ARP32224_P050; AVIVA System Biology, Saan Diego, CA, USA) at 5 μg/ml, anti-Col2 (#7050; Chondrex, WA, USA) at 1:500 (vol/vol) dilution, anti-Sox9 (#AMAB90759; Sigma) at 1:200 and anti-Col10 (LSL LB-0092; Cosmo Bio, Tokyo, Japan) at 1:500 (vol/vol) dilution. Immunoreactivity was visualized with Alexa Fluor® 555 dye-conjugated anti-rabbit IgG (Invitrogen, CA, USA), and counterstaining was performed using 4′,6-diamidino-2-phenylindole, in accordance with the manufacturer’s protocol.

### In situ hybridization

The protocol for in situ hybridization has been described in a previous report^53^. Briefly, tissues harvested from WT and *Dmrt2*^*−/−*^ littermate mice were fixed with 4% paraformaldehyde and then embedded in paraffin. The tissue blocks were cut into 4-μm-thick sections. Digoxigenin (DIG)-11-UTP-labeled, single-stranded RNA probes were prepared using a DIG RNA labeling kit (Roche), in accordance with the manufacturer’s instructions. We used a 0.4 kb fragment of mouse *Col2a1* cDNA, a 0.65 kb fragment of mouse *Col10a1* cDNA, and a 0.57 kb fragment of mouse *Ihh* cDNA to generate antisense and sense probes. Signals were detected with an alkaline phosphatase-conjugated anti-DIG antibody (Roche). All probes were kindly provided by Dr. Noriyuki Tsumaki (Kyoto University, Kyoto, Japan).

### BrdU assay

Pregnant mice were anesthetized and administered 1 ml of BrdU Labeling Reagent (Life Technologies) per 100 g body weight by intraperitoneal injection. The mice were sacrificed 2 h later, after which E17.5 WT and *Dmrt2*^*−/−*^ littermate mouse tibiae were collected and fixed with 4% paraformaldehyde. Paraffin-embedded sections were subjected to a BrdU assay using a BrdU Staining kit (Invitrogen). For quantitative analysis, BrdU-positive nuclei in round and hypertrophic chondrocytes were excluded.

### Western blotting

Cells were rinsed twice with PBS and solubilized in lysis buffer [20 mM HEPES, pH 7.4, 150 mM NaCl, 1 mM EGTA, 1.5 mM MgCl_2_, 10% glycerol, 1% Triton X-100, 10 μg/ml aprotinin, 10 μg/ml leupeptin, 1 mM 4-(2-aminoethyl) benzenesulfonyl fluoride hydrochloride, and 0.2 mM sodium orthovanadate]. The lysates were centrifuged at 4 °C for 10 min at 15,000 × *g* and then boiled in sodium dodecyl sulfate (SDS) sample buffer containing 0.5 M β-mercaptoethanol for 5 min. The supernatant was separated by SDS-polyacrylamide gel electrophoresis, transferred to a nitrocellulose membrane, immunoblotted with a primary antibody, and then visualized with horseradish peroxidase-coupled anti-mouse or -rabbit IgGs using an enhanced chemiluminescence detection kit (Immunostar LD; WAKO, Osaka, Japan). An anti-Flag (M2, dilution ratio 1:2000) antibody was purchased from Sigma-Aldrich. Anti-HA (Y-11, #sc-805, dilution ratio 1:1000) antibody was purchased from Santa Cruz Biotechnology (CA, USA). Antibodies against β-actin (M177-3, dilution ratio 1:1000) and Runx2 (D1L7F, dilution ratio 1:1000) were purchased from MBL (Nagoya, Japan) and CST (MA, USA), respectively. Uncropped images of western blotting are included in Supplementary Fig. 10.

### Whole-mount in situ hybridization

DIG-labeled antisense RNA probes for *Col2a1* and *Sox9* were generated with SP6 or T7 RNA polymerase using the DIG RNA Labeling kit, in accordance with the manufacturer’s instructions. Embryos of WT and *Dmrt2*^*−/−*^ littermate mice at E13.5 were fixed in 4% paraformaldehyde/PBS overnight at 4 °C. The samples were then hybridized overnight with gene-specific DIG-labeled RNA probes at 70 °C overnight. Then, the samples were washed and incubated with an anti-DIG antibody (Antidigoxigenin-AP; Roche). Nitroblue tetrazolium and BCIP (5-bromo-4-chloro-3′-indolylphosphate p-toluidine) were used for signal detection.

### ChIP assays

ChIP analysis was performed using a ChIP-IT Express kit (Active Motif), in accordance with the manufacturer’s instructions. Briefly, primary chondrocytes and ATDC5 cells were washed with PBS, and chromatin was fixed with formaldehyde to crosslink the proteins to chromatin. Crosslinked chromatin was sonicated with Covaris M220. Sonicated chromatin was immunoprecipitated with an anti-H3K27ac antibody (D5E4, CST), anti-Flag (M2) antibody, and anti-Runx2 antibody (D1L7F, CST). DNA fragments were precipitated with protein-A magnetic beads and amplified by PCR using primer pairs specific for the Sox9-bound enhancer of the *Dmrt2* gene (sense primer, 5′-TTCCAGATGGGCTGAAAC-3′; anti-sense primer, 5′-CTGTGCATTGTGGGAGAG-3′), *Ihh* gene promoter containing the Dmrt2-binding element (sense primer, 5′-TGGCCTTTCTCCCTTGTTTTT-3′; anti-sense primer, 5′-CAATGTAGCCTTGTCAGGAGTCA-3′), and *Ihh* gene promoter containing the Runx2-binding element (sense primer, 5′-AGCTTTCGGGTTTGCTTCTC-3′; anti-sense primer, 5′-GTCTCTCCTTCCCGTTCCTT-3′). Quantitative analysis of ChIP assays was performed by real-time PCR using SYBR Green.

### DNA pulldown assays

Cells were lysed in lysis buffer (20 mM HEPES, pH 7.4, 150 mM NaCl, 1 mM EGTA, 1.5 mM MgCl_2_, 10% glycerol, 1% Triton X-100, 10 μg/ml leupeptin, 1 mM PMSF, and 0.2 mM sodium orthovanadate). Lysates pre-incubated with streptavidin agarose beads were incubated with 1 μg of a biotinylated double-stranded oligonucleotide probe containing the Dmrt2-binding element in the *Ihh* gene promoter (sense primer, 5′-AAATAACCAAGATACAATTTGCAAAACACA-3′; anti-sense primer, 5′-TGTGTTTTGCAAATTGTATCTTGGTTATTT-3′) for 3 h. Precipitated oligonucleotides were collected with streptavidin magnetic beads (Dynabeads My One Streptavidin T1; Invitrogen) and washed with lysis buffer. The magnetic beads were resuspended with SDS sample buffer, boiled for 5 min, and then subjected to western blotting.

### Statistics and reproducibility

Randomization and blinding were not performed in the animal studies. Sample sizes were estimated based on previous studies of endochondral bone formation^44,53^. Data were statistically analyzed by unpaired Student’s *t* test for comparison between two groups. For more than two groups, we used one-way analysis of variance (ANOVA) or two-way ANOVA followed by Tukey’s multiple comparisons test. At least five mice (*n* = 5–6) were used for the phenotypic analysis. *P* values of less than 0.05 were considered statistically significant. All results including RT-qPCR and western blotting were performed two or three times independently and reproduced with similar results.

## Supplementary information

Supplementary Information

Description of Additional Supplementary Files

Supplementary Data 1

## Data Availability

RNA-seq data that support the findings of this study have been deposited in the NCBI Gene Expression Omnibus (https://www.ncbi.nlm.nih.gov/geo/) with the accession code GSE155118. Source Data can be found in Supplementary Data 1. All other data are available from the corresponding author on reasonable request.
