# Supplementary material for: Dmrt2 promotes transition of endochondral bone formation by linking Sox9 and Runx2
Source: Commun Biol. 2021 Mar 11;4:326. doi: 10.1038/s42003-021-01848-1 (PMC7952723; doi:10.1038/s42003-021-01848-1)
Supplement: Supplementary file 1 — Supplementary Information [file 42003_2021_1848_MOESM1_ESM.pdf]

Supplementary Table 1

Enrichment FDR	Genes in list	Total genes	Functional Category	Genes
2.41E-09	160	1764	Molecular function regulator	SEMA4F CCNE1 TBC1D8 IL11 BMP7 CCL9 SEMA6A LLGL2 SEMA4D SEMA3G FETUB CXCL13 TSLP IL33 BMP2 NGF IL6RA SEMA4A CCNE2 CXCL5 CXCL15 EREG CXCL3 CXCL1 NPY TGFA COX6A2 RENBP LIF TBC1D10A CXCL10 CCL5 CCL7 CCL2 ARHGAP26 GDF5 SEMA7A GDF15 GRTP1 BMP6 USP6NL PREX1 SAA4 SAA3 PPP1R15A OSGIN2 ATP1B2 PREB INHBE SCN3B STFA3 BIRC2 CXCL2 FXYD2 SERPINA3C SERPINA3F SAA1 SERPINA3M SERPINA3I WNT7B ANGPTL4 IL12B SLPI IL6 FN1 RGS16 TESC TRIB1 FGF2 RAP1GAP2 DOCK8 PRKCB FRY S100A7A 2610528A11RIK WNT3 SIPA1L2 TIAM1 JAG2 DENND2C PCOLCE2 WNT11 ARFGEF3 STC2 RGS9 WARS NAIP1 RASGRF2 ARHGEF3 ITIH4 GDNF SLURP1 C3 CST6 AFAP1L2 NGEF ATP2B4 NCF2 SPINT1 PDYN ANGPT4 EDN3 ARHGEF16 PRKCZ PPP2R2C RASAL1 GPNMB A2M RASGEF1A FGF21 RGS10 TRF FGD5 ADRB1 TNFSF9 DENND3 WNT4 ARHGEF26 PSD ARHGEF4 CDKN1C PPP1R16B DOCK10 PTPN3 MTMR12 DOCK3 WFDC5 KCNS1 CMTM5 RAP1GAP RAPGEF5 WFDC12 ARHGEF37 ADRB2 PPP1R3B PTHLH IL17D LGALS3 FOXL2 PPP1R36 OSTN TG ADM2 FAM19A5 SPINK5 BCL2 FNIP2 PPP1R1B PPP1R3C FGFR1OP SPINT2 NAIP2 MIA KCNIP3 SEC14L2 NOS2 MMP25 BAG1 CD24A RGS5
3.88E-09	149	1628	Signaling receptor binding	WNT3 LCK SEMA4F ICOSL IL12RB1 HCK IL11 SH2B2 HAP1 BMP7 WNT11 CCL9 SEMA6A ITGB4 TRAF3 SEMA4D PTCH1 SEMA3G WNT7B CXCL13 TSLP GNAL GNA14 IL33 BMP2 NGF IL6RA EFNA1 SEMA4A CNTFR CXCL5 CXCL15 EREG CXCL3 CXCL1 GPNMB NPY TGFA WNT5B NLGN3 LIF GNA15 CXCL10 CCL5 CCL7 CCL2 TNFSF9 WNT4 ICAM1 SHANK2 GDF5 SEMA7A GDF15 IRS2 BMP6 SAA4 SAA3 CBLC OSGIN2 PILRA INHBE DLG2 IRS4 CXCL2 PILRB2 PILRB1 SAA1 IL12B LAMA5 LBP IL6 CHAC1 FGF2 OSTN S100A7A 2610528A11RIK TIAM1 SLC9A3R2 JAG2 PROX1 C1QBP STC2 ERN1 NOS2 LCP1 GDNF COL2A1 SLURP1 C3 BAMBI PTPN2 CD74 JAK2 SORBS1 SLIT1 FN1 LRP4 PDYN ANGPT4 EDN3 TLR2 RSPO1 ARHGEF16 PXN A2M FGF21 F7 FAM83B S100B PPARGC1B GRIP1 ADRB1 TGFBI MAG ENPP1 CMTM5 ADORA1 HILPDA PTHLH IL17D LGALS3 OPRD1 GPR17 PRKCB TG ADM2 CADM4 RND1 FAM19A5 CEBPB CD2AP PPP1R1B KDR MIA DHH NGEF LRG1 FOXL2 CEACAM1 CXADR DBI PDPN ATP2B2 TRF MST1 ADRB2 MMP13 PPID ADAM8
6.77E-08	34	191	Glycosaminoglycan binding	ADGRG1 CRISPLD2 EVA1C COLQ PTCH1 GPNMB EGFLAM RTN4R NAV2 BCAN FGFR4 HAPLN4 FGFRL1 BMP7 PCOLCE2 C1QBP HAPLN1 FN1 COL11A1 TLR2 HAPLN3 ACAN CXCL10 CCL7 CCL2 FGF2 PLA2G5 LIPH NOD2 SAA1 SUSD5 CXCL13 CCL5 COL23A1
6.77E-08	62	501	Receptor ligand activity	SEMA4F IL11 BMP7 CCL9 SEMA6A SEMA4D SEMA3G CXCL13 TSLP IL33 BMP2 NGF IL6RA SEMA4A CXCL5 CXCL15 EREG CXCL3 CXCL1 NPY TGFA LIF CXCL10 CCL5 CCL7 CCL2 GDF5 SEMA7A GDF15 BMP6 SAA4 SAA3 OSGIN2 INHBE CXCL2 SAA1 WNT7B IL12B IL6 FGF2 S100A7A 2610528A11RIK WNT3 JAG2 STC2 GDNF SLURP1 PDYN EDN3 GPNMB FGF21 TNFSF9 WNT4 CMTM5 PTHLH IL17D LGALS3 OSTN TG ADM2 FAM19A5 MIA
2.75E-07	63	536	Receptor regulator activity	SEMA4F IL11 BMP7 CCL9 SEMA6A SEMA4D SEMA3G CXCL13 TSLP IL33 BMP2 NGF IL6RA SEMA4A CXCL5 CXCL15 EREG CXCL3 CXCL1 NPY TGFA LIF CXCL10 CCL5 CCL7 CCL2 GDF5 SEMA7A GDF15 BMP6 SAA4 SAA3 OSGIN2 INHBE CXCL2 SAA1 WNT7B IL12B IL6 FGF2 S100A7A 2610528A11RIK WNT3 JAG2 STC2 GDNF SLURP1 PDYN ANGPT4 EDN3 GPNMB FGF21 TNFSF9 WNT4 CMTM5 PTHLH IL17D LGALS3 OSTN TG ADM2 FAM19A5 MIA
7.05E-07	53	427	Metal ion transmembrane transporter activity	GRIK3 ATP7B MCOLN2 TRPV4 TRPV2 SLC6A15 SLC39A14 SLC11A2 KCNK5 SLC18A2 SLC9A2 ATP2B4 SLC4A10 KCNQ5 SLC30A2 SLC34A2 ITPR2 ATP2B2 GRIK4 KCNK1 ATP2C2 MCOLN3 SLC9A7 SLC12A9 KCNA7 ABCC8 KCNS1 ATP1B2 SLC39A11 JPH1 KCNG4 KCNK12 SLC39A8 KCNC1 ASIC2 SLC1A1 SLC8A3 NIPAL4 SLC6A19 TFRC SLC6A9 SLC6A12 TRPM1 TRF ASIC4 KCNJ6 KCNJ4 SCN3B KCNC3 SLC4A11 KCNIP3 HPN CPOX
8.56E-07	98	1032	Transmembrane transporter activity	GRIK3 ABCB1A CLCN3 ATP7B SLC38A3 MCOLN2 TRPV4 CLIC3 TRPV2 SLC6A15 ABCA8B SLC25A29 SLC6A19 SLC39A14 SLC11A2 KCNK5 SLC18A2 MIP PIDD1 SLC9A2 SLC16A14 ATP2B4 SLC4A10 CHRNA4 KCNQ5 SLC35D1 SLC6A9 SLC30A2 ABCB1B SLC25A33 SLC34A2 P2RX7 ITPR2 ATP2B2 COX6A2 SLC7A3 SLC7A2 UCP1 GRIK4 SLCO2A1 ABCC4 SLC16A1 KCNK1 ATP2C2 TMEM63C SLC26A2 MCOLN3 SLC9A7 SLC12A9 KCNA7 SLC19A3 ABCC8 KCNS1 SLC7A1 ATP1B2 SLC39A11 JPH1 KCNG4 SLC26A1 SLC35C1 KCNK12 SLC39A8 PANX2 KCNC1 SLC35E3 SLC22A4 ASIC2 SLC1A1 SLC2A1 TRPM1 ANO1 GJB2 SLC8A3 SLC1A5 NIPAL4 TFRC SLC37A1 SLC52A3 SLC35A3 SLC6A12 SLC37A2 TRF ASIC4 SLC35F1 SLC35F2 SLC26A9 KCNJ6 KCNJ4 SCN3B SLC25A40 BCL2 KCNC3 SLC22A14 SLC4A11 KCNIP3 HPN CEACAM1 CPOX

1.53E-06	125	1443	Protein dimerization activity	CDH4 SHMT1 BCL2 HIP1R IL12B ODC1 RRM2 ERN1 NOS2 EPAS1 SOX8 CIDEA LRP4 TPD52 TESC BHLHE41 CBLN1 HEYL PDSS2 GDF15 SOX5 PTPRE DMRTA1 DMRT2 CEBPB JMJD6 CEBPD BNIP3 FBXO7 CLCN3 HIF3A TFAP4 TCF23 TWIST2 ASCL2 RAB11FIP4 STK4 BTBD11 STK10 STC2 FLT4 GRHL1 NPAS3 FOS WARS AHRR HMGCR CRYL1 GDNF MYC CPOX TFRC ACHE IMPA2 NUDT5 NPAS2 ATF3 LCN2 BMP2 CHMP4C NTSR1 IL6RA TLR2 MTTP RRAGD TFAP2C GALE CDA PRKG2 PSPH NUP210 CRYM ANO1 STK26 RENBP ABTB2 SLC16A1 S100B UPB1 CCL5 ADRB1 ENPP1 GCH1 BMP6 HEY1 RABGGTA ADORA1 S100A1 ADRB2 IL17D BHLHA15 TAF4B CAMK2B H2AFJ FGFR1OP SLC4A11 GBP5 KCNIP3 DMBX1 MAG E2F8 SOX6 JAM2 PDXP CKMT1 GGCT AMPH CXCL13 CD28 DBI CHRNA4 MTUS2 A2M HP FAAH INA RIMS2 MTHFD1L RAP1GAP DLG2 PTPRT TG PANX2 COL23A1 CEACAM1
2.66E-06	107	1192	Transporter activity	GRIK3 ABCB1A CLCN3 ATP7B SLC38A3 MCOLN2 TRPV4 CLIC3 TRPV2 SLC6A15 ABCA8B SLC25A29 SLC6A19 SLC39A14 SLC11A2 KCNK5 SLC27A6 SLC18A2 ATP10A MIP PIDD1 SLC9A2 SLC16A14 ATP2B4 SLC4A10 CHRNA4 KCNQ5 MTTP SLC35D1 SLC6A9 SLC30A2 ABCB1B SLC25A33 SLC34A2 P2RX7 ITPR2 ATP2B2 COX6A2 SLC7A3 SLC7A2 UCP1 GRIK4 SLCO2A1 ABCC4 SLC16A1 KCNK1 ATP2C2 TMEM63C SLC26A2 MCOLN3 SLC9A7 SLC12A9 KCNA7 SLC19A3 ABCC8 KCNS1 RAMP3 SLC7A1 ATP1B2 SLC39A11 JPH1 OSBPL1A KCNG4 SLC26A1 SLC35C1 KCNK12 SLC39A8 ATP10B PANX2 KCNC1 SLC35E3 SLC22A4 ASIC2 SLC1A1 SLC2A1 TRPM1 ANO1 GRAMD1C GJB2 SLC8A3 SLC1A5 NIPAL4 TFRC SLC17A9 SLC37A1 SLC52A3 SLC35A3 SLC6A12 C2CD2L SLC37A2 TRF ASIC4 SLC35F1 SLC35F2 SLC26A9 KCNJ6 KCNJ4 SCN3B SLC25A40 BCL2 KCNC3 SLC22A14 SLC4A11 KCNIP3 HPN CEACAM1 CPOX
3.32E-06	66	622	Cation transmembrane transporter activity	GRIK3 CLCN3 ATP7B SLC38A3 MCOLN2 TRPV4 TRPV2 SLC6A15 SLC25A29 SLC39A14 SLC11A2 KCNK5 SLC18A2 SLC9A2 ATP2B4 SLC4A10 KCNQ5 SLC6A9 SLC30A2 SLC34A2 P2RX7 ITPR2 ATP2B2 COX6A2 SLC7A3 SLC7A2 GRIK4 KCNK1 ATP2C2 TMEM63C MCOLN3 SLC9A7 SLC12A9 KCNA7 ABCC8 KCNS1 SLC7A1 ATP1B2 SLC39A11 JPH1 KCNG4 KCNK12 SLC39A8 KCNC1 SLC22A4 ASIC2 SLC1A1 TRPM1 SLC8A3 SLC1A5 NIPAL4 SLC6A19 TFRC CHRNA4 SLC6A12 ANO1 TRF ASIC4 KCNJ6 KCNJ4 SCN3B KCNC3 SLC4A11 KCNIP3 HPN CPOX
4.67E-06	78	793	Inorganic molecular entity transmembrane transporter activity	GRIK3 CLCN3 ATP7B SLC38A3 MCOLN2 TRPV4 CLIC3 TRPV2 SLC6A15 SLC6A19 SLC39A14 SLC11A2 KCNK5 SLC18A2 MIP PIDD1 SLC9A2 SLC16A14 ATP2B4 SLC4A10 CHRNA4 KCNQ5 SLC6A9 SLC30A2 SLC34A2 P2RX7 ITPR2 ATP2B2 COX6A2 SLC7A3 SLC7A2 GRIK4 SLCO2A1 SLC16A1 KCNK1 ATP2C2 TMEM63C SLC26A2 MCOLN3 SLC9A7 SLC12A9 KCNA7 ABCC8 KCNS1 SLC7A1 ATP1B2 SLC39A11 JPH1 KCNG4 SLC26A1 KCNK12 SLC39A8 KCNC1 ASIC2 SLC1A1 TRPM1 ANO1 SLC8A3 SLC1A5 NIPAL4 SLC25A29 TFRC SLC37A1 SLC6A12 SLC37A2 TRF ASIC4 SLC26A9 KCNJ6 KCNJ4 SCN3B KCNC3 SLC4A11 KCNIP3 HPN ABCC4 CEACAM1 CPOX
9.62E-06	81	851	Ion transmembrane transporter activity	GRIK3 CLCN3 ATP7B SLC38A3 MCOLN2 TRPV4 CLIC3 TRPV2 SLC6A15 SLC25A29 SLC6A19 SLC39A14 SLC11A2 KCNK5 SLC18A2 PIDD1 SLC9A2 SLC16A14 ATP2B4 SLC4A10 CHRNA4 KCNQ5 SLC35D1 SLC6A9 SLC30A2 SLC34A2 P2RX7 ITPR2 ATP2B2 COX6A2 SLC7A3 SLC7A2 GRIK4 SLCO2A1 SLC16A1 KCNK1 ATP2C2 TMEM63C SLC26A2 MCOLN3 SLC9A7 SLC12A9 KCNA7 ABCC8 KCNS1 SLC7A1 ATP1B2 SLC39A11 JPH1 KCNG4 SLC26A1 KCNK12 SLC39A8 KCNC1 SLC22A4 ASIC2 SLC1A1 SLC2A1 TRPM1 ANO1 SLC8A3 SLC1A5 NIPAL4 TFRC SLC37A1 SLC52A3 SLC6A12 SLC37A2 TRF ASIC4 SLC26A9 KCNJ6 KCNJ4 SCN3B KCNC3 SLC4A11 KCNIP3 HPN ABCC4 CEACAM1 CPOX
1.12E-05	148	1868	Identical protein binding	CDH4 SHMT1 FTH1 SHMT2 BCL2 HIP1R IL12B ODC1 RRM2 ERN1 NOS2 CIDEA LRP4 TPD52 TESC CBLN1 HEYL CCDC155 GDF15 PTPRE DMRTA1 DMRT2 CEBPB JMJD6 C1QTNF3 CEBPD BNIP3 LCK ANGPTL4 HAS1 NQO1 SLBP SH2B2 TFAP4 SOD2 TRPV4 SDC4 RAB11FIP4 MMP9 STK4 SULT4A1 TNFAIP3 STK10 STC2 FLT4 SDC1 GRHL1 NFKBIA WARS FBP2 HMGCR CRYL1 GDNF CPOX TFRC ACHE IMPA2 JAK2 NUDT5 FN1 ATF3 MCM10 OPTN LCN2 CHMP4C NTSR1 IL6RA TLR2 CASP6 CTH ALDOB HOOK1 SLC2A1 GALE CDA PRKG2 PSPH HOXA1 M1AP CRYM STK26 RENBP DCTD UBASH3B ETS1 SLC16A1 S100B UPB1 KCNK1 CCL5 PPFIBP2 ENPP1 GCH1 CISD1 GDF5 FOXO4 RASEF S100A1 ADRB2 GJB2 FOXC2 IL17D GP1BB SKP2 FGFR3 BIRC2 CAMK2B KDR FGFR1OP FBP1 GBP5 KCNIP3 RELB LCP1 COL2A1 DMBX1 BHLHE41 ANO1 MAG E2F8 BHLHA15 SYT9 PDXP CKMT1 ICOSL GGCT HAP1 TRPV2 PFKP SEMA4D CXADR CD28 RASSF5 BMP2 MTUS2 A2M BCAT1 HP FAAH MTHFD1L RAP1GAP PTPRT TG COL23A1 CEACAM1 C1S2 SDK1 CLDN12
1.12E-05	85	914	Protein homodimerization activity	CDH4 SHMT1 BCL2 HIP1R IL12B ODC1 RRM2 ERN1 NOS2 CIDEA LRP4 TPD52 TESC CBLN1 HEYL GDF15 PTPRE DMRTA1 DMRT2 CEBPB JMJD6 CEBPD BNIP3 TFAP4 RAB11FIP4 STK4 STK10 STC2 FLT4 GRHL1 WARS HMGCR CRYL1 GDNF CPOX TFRC ACHE IMPA2 NUDT5 ATF3 LCN2 CHMP4C NTSR1 IL6RA GALE CDA PRKG2 PSPH CRYM STK26 RENBP SLC16A1 S100B UPB1 CCL5 ENPP1 GCH1 S100A1 ADRB2 IL17D CAMK2B FGFR1OP GBP5 KCNIP3 DMBX1 BHLHE41 ANO1 MAG E2F8 BHLHA15 PDXP CKMT1 GGCT CD28 BMP2 MTUS2 A2M HP FAAH MTHFD1L RAP1GAP PTPRT TG COL23A1 CEACAM1
1.46E-05	24	140	Heparin binding	ADGRG1 EVA1C COLQ PTCH1 GPNMB CRISPLD2 RTN4R NAV2 FGFR4 FGFRL1 BMP7 PCOLCE2 FN1 COL11A1 CXCL10 CCL7 CCL2 FGF2 PLA2G5 LIPH SAA1 CXCL13 CCL5 COL23A1

1.72E-05	209	2876	Anion binding	HIP1R CLCN3 SHMT1 NOS2 PFKP AMPH ANXA11 GOLPH3 SYT7 SHMT2 TUBA4A SOAT1 CTH ITPR2 ADGRG1 GCH1 ESYT3 EVA1C CYB5R2 PLEKHF2 RND1 COLQ SYT17 SYT9 WDR35 GSDMC CRABP2 DYRK3 PLEK2 PTCH1 GPNMB MYH14 UCP1 CRISPLD2 RAB27A ADGRB1 MAG KDM1B MREG RTN4R DOK7 NAV2 CKMT1 LCK HK2 MCM2 HCK BCAN RIPK4 FGFR4 MCM5 ATP7B HAPLN4 RAD51C FGFRL1 BMP7 GM266 STEAP4 TRPV4 PCOLCE2 STK4 C1QBP DNAH11 P4HA2 RPS6KL1 STK10 CDC34 SLC22A4 FLT4 ABCA8B ERN1 RAB37 WARS KIF26A PXDC1 HAPLN1 NAIP1 HMGCR CRYL1 PLA2G7 EHD3 IP6K3 MAP3K8 MYO7B GNAL GNA14 JAK2 PIP5K1B PAPSS2 SLC1A1 HSPA12A GOT1 ATP10A FAM20C ARL10 FN1 RAB17 DBI NR5A2 ATP2B4 UCK2 PHYH PRKCQ LCN2 STK39 TNIK NPR1 COL11A1 LRAT RRAGD GNE AK4 ORC1 ABCB1B PRKCZ PI4K2B SLC34A2 PRKG2 P2RX7 RASAL1 RET CAMK1 ATP2B2 HAPLN3 ACAN FCHSD2 ACSM3 STK26 PIM2 RAB39B RENBP RAB20 GPT2 JAK3 MAP3K21 C2CD2L NMNAT3 DCLK3 TRIB1 ABCC4 SNAP91 FAM20B RECQL4 ATP2C2 RASD2 GNA15 CXCL10 CCL7 CCL2 PLK5 ZRANB3 ARHGAP26 DNA2 PSD ZFYVE28 FGF2 ENPP1 SNX22 PREX1 GOT1L1 STK32A ABCC8 CDK6 ABCB1A PPIP5K2 MTHFD1L PLA2G5 CHD7 STX3 MCM3 RASEF DIRAS1 LIPH BMPR1B PMP2 PRKCB TDRD9 FGFR3 ATP10B NOD2 CAMK2B ITPK1 MDN1 SYN3 DGKK KDR FBP1 ETNK2 MYO18B SAA1 GRAMD2 TTLL9 NLRP12 NAIP2 MTHFSL SUSD5 GBP5 PDE1C PTGDS CXCL13 SLIT1 ALDOB FAAH CCL5 BDH1 TG COL23A1 DAB1 VNN1
2.23E-05	69	706	Calcium ion binding	CDH4 ANXA11 ENPP2 SYT7 SPARCL1 ITPR2 ESYT3 SYT17 SYT9 ADAM8 FAM20C TESC S100A3 SCIN JAG2 BCAN CLSTN3 CDH23 MATN4 RAB11FIP4 MATN3 EFCAB11 LPCAT1 CDHR1 LCP1 DHH PCDHGC4 EHD3 DSC2 SLIT1 PLCD4 GALNT3 PRRG4 PAMR1 LRP4 NECAB3 TPD52 PLA2G12A LRP8 HSPG2 CGREF1 PSPH RET ACAN F7 CLSTN2 LPCAT2 S100B FSTL4 GCH1 C1S1 SCUBE3 CBLC MATN1 EFHD2 PLA2G5 EGFLAM RASEF S100A1 DSG2 CCBE1 GJB2 MMP13 PCDH1 S100A7A EFCAB1 KCNIP3 C1S2 ATP2B2
2.82E-05	59	574	Inorganic cation transmembrane transporter activity	GRIK3 CLCN3 ATP7B MCOLN2 TRPV4 TRPV2 SLC6A15 SLC39A14 SLC11A2 KCNK5 SLC18A2 SLC9A2 ATP2B4 SLC4A10 KCNQ5 SLC30A2 SLC34A2 P2RX7 ITPR2 ATP2B2 COX6A2 GRIK4 KCNK1 ATP2C2 TMEM63C MCOLN3 SLC9A7 SLC12A9 KCNA7 ABCC8 KCNS1 ATP1B2 SLC39A11 JPH1 KCNG4 KCNK12 SLC39A8 KCNC1 ASIC2 SLC1A1 TRPM1 SLC8A3 NIPAL4 SLC6A19 TFRC CHRNA4 SLC6A9 SLC6A12 ANO1 TRF ASIC4 KCNJ6 KCNJ4 SCN3B KCNC3 SLC4A11 KCNIP3 HPN CPOX
4.72E-05	16	74	Antiporter activity	CLCN3 SLC9A2 SLC35D1 SLC26A2 SLC9A7 SLC26A1 SLC35C1 SLC35E3 SLC4A10 SLC8A3 SLC38A3 SLC22A4 SLC37A1 SLC37A2 SLC26A9 SLC4A11
0.000121983	12	46	Acetylgalactosaminyltransferase activity	B3GNT5 B3GNT7 GALNT7 GALNT6 B4GALNT2 GALNT14 GALNT3 CSGALNACT1 GALNT12 B4GALNT3 B4GALNT4 GALNT2
0.000166713	66	738	DNA-binding transcription factor activity, RNA polymerase II-specific	SPI1 RELB ELF3 HIF3A EPAS1 SOX8 OVOL1 NR5A2 LMX1A LHX6 TFAP2C LHX5 KLF15 BARX2 ETS1 GBX2 FOXA2 SP6 IRF8 FOXO4 ZFP367 E2F8 FOXC2 FOXL2 JUNB SOX7 SOX11 ARID3C FOXE1 RHOX12 TFCP2L1 EOMES DMRT2 DLX3 HOXC13 TFAP4 PROX1 RBPJL MYB FOS GLI1 ATF3 DMBX1 BHLHE41 HEYL SOX5 GLI2 NKX3-2 BHLHA15 CEBPB CEBPD KCNIP3 ASCL2 GRHL1 EAF2 FOSL1 BATF3 RORC NKX6-1 PRDM1 FOXF2 ZFP750 HEY1 SOX2 SOX6 PLAGL1 MYC PREB
0.000166713	30	232	Cytokine activity	IL11 BMP7 CCL9 CXCL13 TSLP IL33 BMP2 CXCL5 CXCL15 CXCL3 CXCL1 LIF CXCL10 CCL5 CCL7 CCL2 GDF5 GDF15 BMP6 INHBE CXCL2 IL12B IL6 2610528A11RIK SLURP1 TNFSF9 CMTM5 IL17D FAM19A5 FGF2
0.000166713	40	355	Active transmembrane transporter activity	ABCB1A CLCN3 ATP7B SLC6A15 ABCA8B SLC11A2 SLC18A2 SLC9A2 ATP2B4 SLC4A10 SLC35D1 ABCB1B SLC34A2 ATP2B2 SLCO2A1 ABCC4 ATP2C2 SLC26A2 SLC9A7 SLC12A9 ABCC8 ATP1B2 SLC26A1 SLC35C1 SLC35E3 SLC1A1 SLC8A3 SLC1A5 SLC38A3 SLC22A4 SLC6A19 SLC37A1 SLC16A14 SLC6A9 SLC6A12 SLC37A2 SLC16A1 SLC26A9 SLC4A11 CPOX
0.000223414	67	733	Lipid binding	HIP1R AMPH ANXA11 GOLPH3 SYT7 SOAT1 RORC ITPR2 ESYT3 CAMP OSBPL1A PLEKHF2 CD14 APOL8 SYT17 SYT9 WDR35 GSDMC CRABP2 PTGDS LBP PLEK2 P2RX7 UCP1 ADGRB1 GRAMD1C RLBP1 MREG DOK7 SEC14L2 ALDH1A2 TRPV4 PXDC1 PTCH1 FYB PLA2G7 EHD3 DBI NR5A2 BPIFB1 TLR2 LRAT MTTP BSPRY RASAL1 FCHSD2 C2CD2L SNAP91 ARHGAP26 MAG MCOLN3 PSD ZFYVE28 SNX22 PREX1 STX3 RBP3 RTN4R PMP2 GRAMD2 PDZD8 C3 ALDOB FAAH BDH1 DAB1 VNN1

0.000223414	166	2284	Carbohydrate derivative binding	NOS2 PFKP TUBA4A SOAT1 ADGRG1 CRISPLD2 GCH1 CAMP EVA1C CD14 RND1 COLQ CHIL1 LBP DYRK3 PTCH1 P2RX7 GPNMB MYH14 UCP1 RAB27A ADGRB1 MAG EGFLAM RTN4R NAV2 NOD2 CKMT1 LCK HK2 MCM2 HCK CLCN3 BCAN RIPK4 FGFR4 MCM5 ATP7B HAPLN4 RAD51C FGFRL1 BMP7 GM266 TRPV4 PCOLCE2 STK4 C1QBP DNAH11 RPS6KL1 STK10 CDC34 SLC22A4 FLT4 GFPT2 ABCA8B ERN1 RAB37 WARS KIF26A HAPLN1 NAIP1 COL2A1 EHD3 IP6K3 MAP3K8 MYO7B GNAL GNA14 JAK2 PIP5K1B PAPSS2 HSPA12A ATP10A FAM20C ARL10 FN1 RAB17 DBI ATP2B4 UCK2 PRKCQ FIBCD1 STK39 TNIK NPR1 COL11A1 TLR2 RRAGD GNE AK4 ORC1 CDA ABCB1B PRKCZ PI4K2B PRKG2 RET CAMK1 ATP2B2 HAPLN3 ACAN ACSM3 STK26 PIM2 RAB39B RENBP RAB20 JAK3 MAP3K21 NMNAT3 DCLK3 TRIB1 ABCC4 FAM20B RECQL4 ATP2C2 RASD2 GNA15 CXCL10 CCL7 CCL2 PLK5 ZRANB3 DNA2 FGF2 ENPP1 STK32A ABCC8 CDK6 ABCB1A PPIP5K2 MTHFD1L PLA2G5 CHD7 MCM3 ADORA1 RASEF DIRAS1 LIPH BMPR1B PRKCB TDRD9 FGFR3 ATP10B CAMK2B ITPK1 MDN1 SYN3 DGKK KDR FBP1 ETNK2 MYO18B SAA1 TTLL9 NLRP12 NAIP2 MTHFSL SUSD5 GBP5 PDE1C CXCL13 SLIT1 CCL5 COL23A1 VNN1
0.000345818	29	230	Secondary active transmembrane transporter activity	CLCN3 SLC6A15 SLC11A2 SLC18A2 SLC9A2 SLC4A10 SLC35D1 SLC34A2 SLCO2A1 SLC26A2 SLC9A7 SLC12A9 SLC26A1 SLC35C1 SLC35E3 SLC1A1 SLC8A3 SLC1A5 SLC38A3 SLC22A4 SLC6A19 SLC37A1 SLC16A14 SLC6A9 SLC6A12 SLC37A2 SLC16A1 SLC26A9 SLC4A11
0.000523406	12	54	Chemokine activity	CCL9 CXCL13 CXCL5 CXCL15 CXCL3 CXCL1 CXCL10 CCL5 CCL7 CCL2 CXCL2 2610528A11RIK
0.000523406	18	111	PDZ domain binding	ATP2B4 ATP2B2 TMEM88 CLCN3 MPP2 SLC22A4 LLGL2 ARHGEF16 TBC1D10A ADRB1 KCNJ4 SSTR2 CCDC88C SHISA9 ADGRB1 CXADR DLGAP3 DLG2
0.000533535	34	299	G protein-coupled receptor binding	WNT3 WNT11 CCL9 PTCH1 WNT7B CXCL13 GNAL GNA14 CXCL5 CXCL15 CXCL3 CXCL1 NPY WNT5B GNA15 CXCL10 CCL5 CCL7 CCL2 WNT4 CXCL2 2610528A11RIK C1QBP ITGB4 C3 BAMBI JAK2 EDN3 RSPO1 ADRB1 ADORA1 PPP1R1B ADRB2 SAA1
0.000606454	21	145	Growth factor activity	IL11 NGF IL6RA EREG TGFA LIF OSGIN2 FGF2 JAG2 IL12B BMP7 GDNF IL6 BMP2 CXCL1 FGF21 GDF5 GDF15 BMP6 INHBE MIA

Supplementary Table 2

Sequences of primer pairs used for RT-qPCR

Dmrt2	sense primer:	5'-CCCAAACTGGAAGGCTCTCTG-3'
	anti-sense primer:	5'-CTGGAACCACCTGGTGAACC-3'
	probe:	5'-CCCATCTGCCAGAGGTCCCAGCCT-3'
Col2a1	sense primer:	5'-CCTCCGTCTACTGTCCACTGA-3'
	anti-sense primer:	5'-ATTGGAGCCCTGGATGAGCA-3'
	probe:	5'-TGAGGTTGCCAGCCGCTTCGTCCA-3'
Sox9	sense primer:	5'-CCTTCAACCTTCCTCACTACAGC-3'
	anti-sense primer:	5'-GGTGGAGTAGAGCCCTGAGC-3'
	probe:	5'-CCGCCCATCACCCGCTCGCAATAC-3'
Ihh	sense primer:	5'-GACTCATTGCCTCCCAGAACTG-3'
	anti-sense primer:	5'-CCAGGTAGTAGGGTCACATTGC-3'
	probe:	5'-CCACAGCCAGCCTGGACATCCCGA-3'
Col10a1	sense primer:	5'-GCCAAGCAGTCATGCCTGAT-3'
	anti-sense primer:	5'-GACACGGGCATACCTGTTACC-3'
	probe:	5'-AGCACTGACAAGCGGCATCCCAGA-3'
Alpl	sense primer:	5'-ATCTTTGGTCTGGCTCCCATG-3'
	anti-sense primer:	5'-TTTCCCGTTCACCGTCCAC-3'
	probe:	5'-TGAGCGACACGGACAAGAAGCCCTT-3'
Tcf7	sense primer:	5'- CAGCTTTCTCCACTCTACGAACA-3'
	anti-sense primer:	5'- CTGCCTGAGGTCAGAGAATAAAATC-3'
	probe:	5'-CAGCGGCCTGTGAACTCCTTGCTTCTG-3'
β-actin	sense primer:	5'-TTAATTTCTGAATGGCCCAGGTCT-3'
	anti-sense primer:	5'-ATTGGTCTCAAGTCAGTGTACAGG-3'
	probe:	5'-CCTGGCTGCCTCAACACCTCAACCC-3'

Supplementary Table 2 (continued)

Sequences of primer pairs used for PCR in Supplementary Figure 1

Dmrt1-F	5'-TCAGACCCCGCCTACTACAG-3'
Dmrt1-R	5'-ACACACTGGCTTTGGCTTCT-3'
Dmrt2-F	5'-CCAAACTGGAAGGCTCTCTG-3'
Dmrt2-R	5'-CTGGCTCTCCTTGACCAAAC-3'
Dmrt3-F	5'-CTTCAGCTTGAAAGCCAACC-3'
Dmrt3-R	5'-GGCCAAAGTATTCCTCAGCA-3'
Dmrt4-F	5'ACAAGCATAGCCGCCTAGAA-3'
Dmrt4-R	5'-CTGGAACCAGTCCAGGTGTT-3'
Dmrt5-F	5'-TCATTGAGAGCTTGGCAGCGCCGGAC-3'
Dmrt5-R	5'-GCTGCTGCTGTCGCCGCACCGGGCAC-3'
Dmrt6-F	5'-CTGAGATTCAAGTCTGATCATGTGG-3'
Dmrt6-R	5'-GAAGTGGAGAGCAGAGAGGTAGCC-3'
Dmrt7-F	5'-CCCTAACCTGCCTCACACAT-3'
Dmrt7-R	5'-GGTGGAGCAGAGAGATCCAG-3'
Gapdh-F	5'-ACATCAAGAAGGTGGTGAAGCAGG-3'
Gapdh-R	5'-CTCTTGCTCTCAGATCCTTGCTGG-3'

Supplementary Figure 1

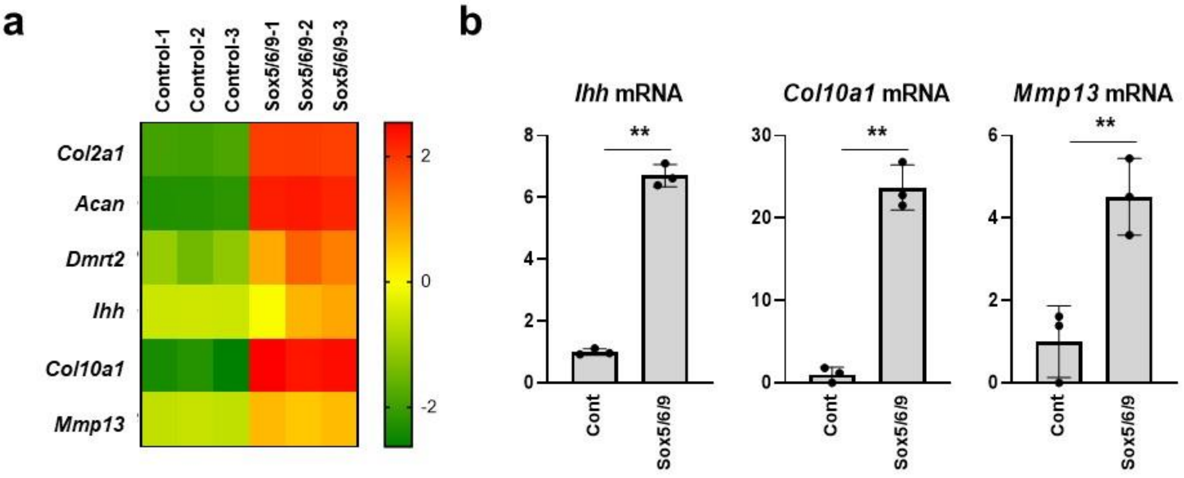

Supplementary Figure 1. Expression pattern of hypertrophic genes in primary chondrocytes overexpressing Sox5/6/9.

(a) Expression heatmap of chondrocyte genes in RNA-seq analysis. This heatmap was created using GraphPad Prism8. (b) Total RNA was isolated from primary chondrocytes infected with control (Cont) or Sox5+Sox6+Sox9 (Sox5/6/9) adenoviruses. *Ihh, Col10a1*, and *Mmp13* mRNA expression levels were analyzed by RT-qPCR. The RNA level is indicated as the fold increase compared with that of the control. Data are shown as the mean ± s.d. (n = 3). **p < 0.01; unpaired Student's t-test.

Supplementary Figure 2

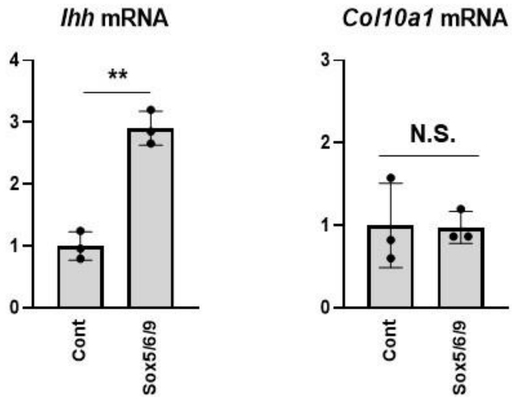

Supplementary Figure 2. Total RNA was isolated from C3H10T1/2 cells infected with control (Cont) or Sox5+Sox6+Sox9 (Sox5/6/9) adenoviruses. *Ihh* and *Col10a1* mRNA expression levels were analyzed by RT-qPCR. The RNA level is indicated as the fold increase compared with that of the control. Data are shown as the mean ± s.d. (n = 3). **$p < 0.01$; unpaired Student's t-test.

Supplementary Figure 3

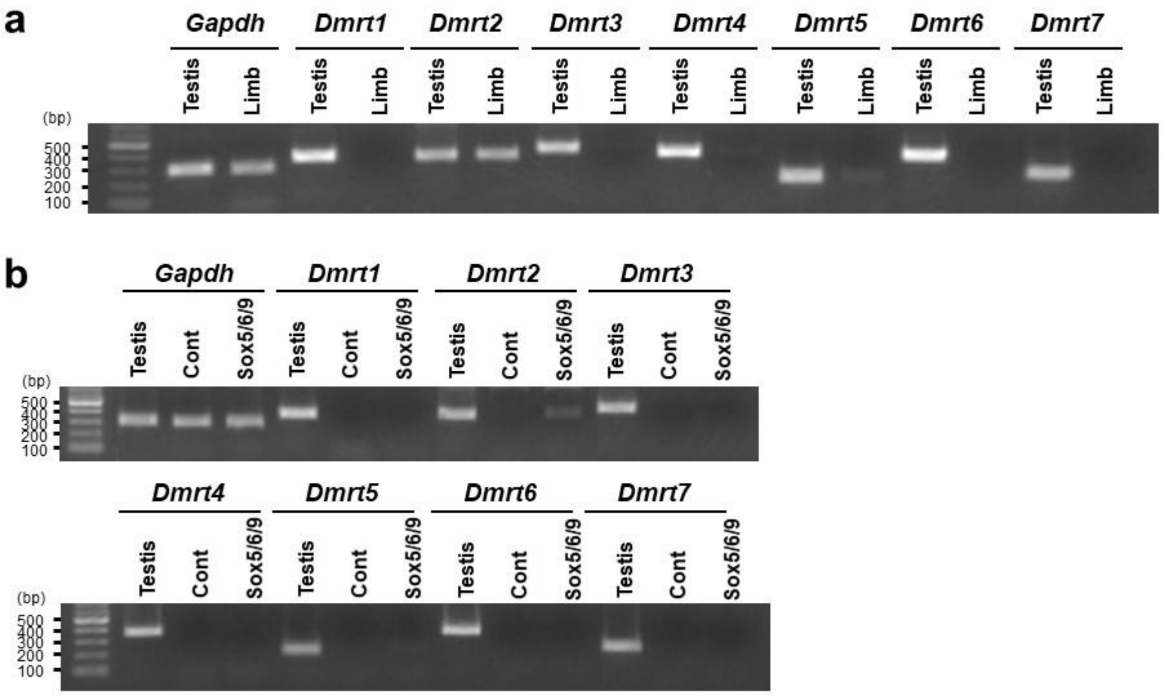

Supplementary Figure 3. Expression of Dmrt family genes in chondrocytes.

(**a**) Total RNA was isolated from testes and limbs of newborn mice, and mRNA expression of Dmrt family genes was determined by RT-PCR. (**b**) Total RNA was isolated from C3H10T1/2 cells infected with control (Cont) or Sox5/6/9 adenoviruses, and the mRNA expression of Dmrt family genes was determined by RT-PCR. Total RNA from the testis was used as a positive control.

Supplementary Figure 4

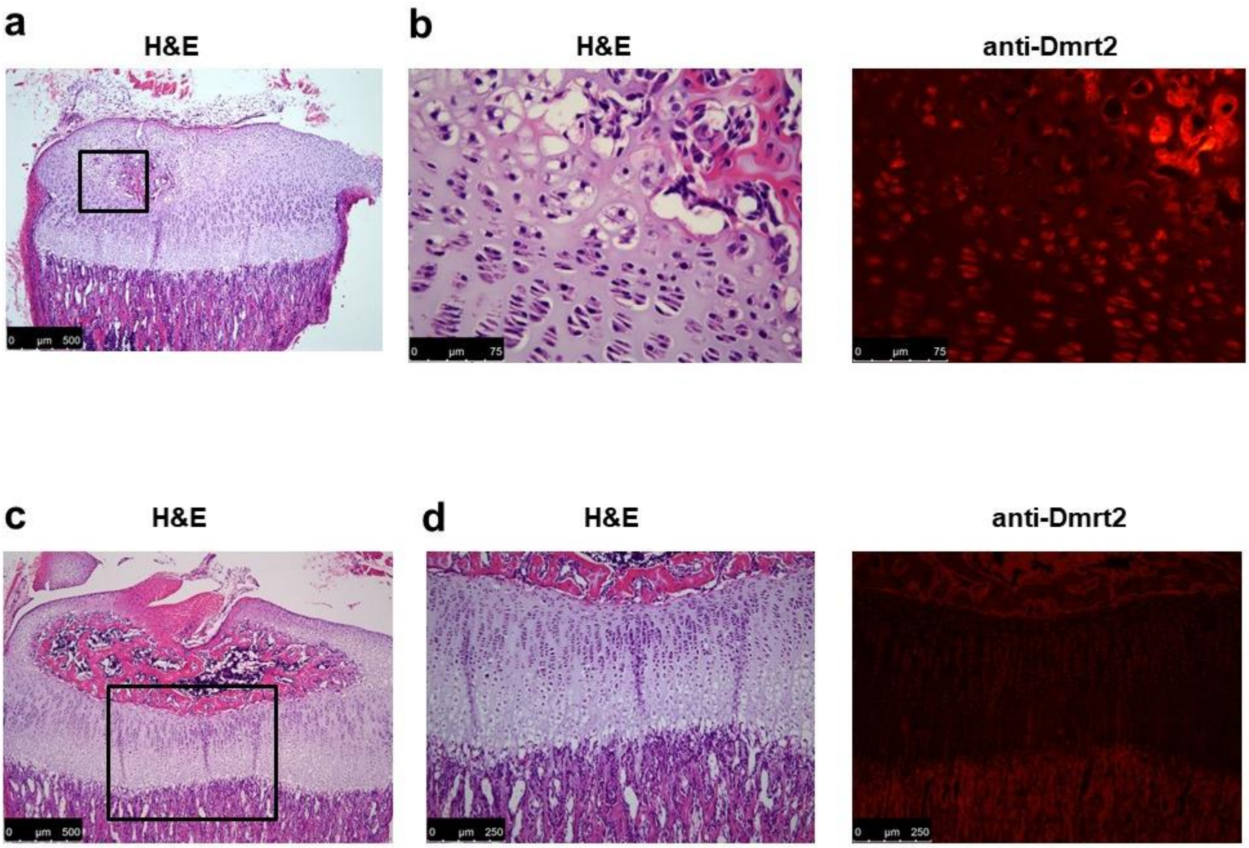

Supplementary Figure 4. Immunohistochemical analysis of Dmrt2 postnatally.
(**a, b**) Sections of 2-week-old male mouse tibia were subjected to H&E staining and immunohistochemical analyses using anti-Dmrt2 antibodies. Higher-magnification images of secondary ossification center (boxed area) are shown in (b). Scale bar: 75 μm. (**c, d**) Sections of 4-week-old male mouse tibia were subjected to H&E staining and immunohistochemical analyses using anti-Dmrt2 antibodies. Higher-magnification images of growth plate (boxed area) are shown in (d). Scale bars: (c) 500 μm and (d) 250 μm.

Supplementary Figure 5

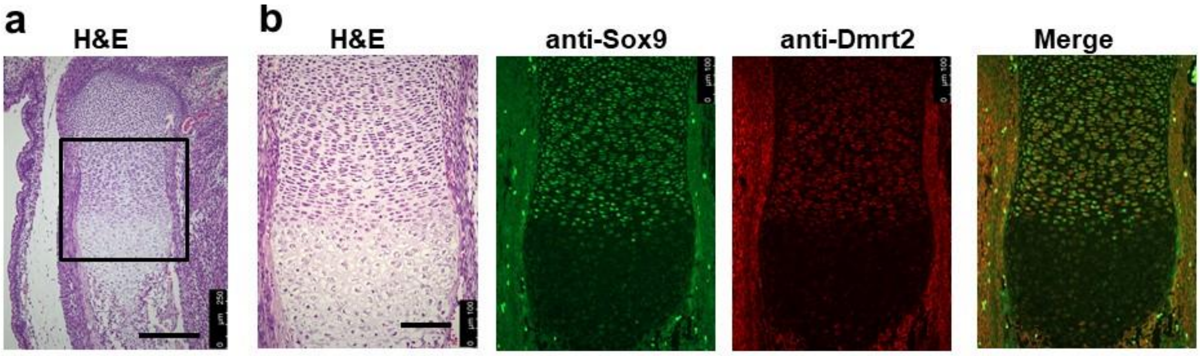

Supplementary Figure 5. Immunohistochemical analysis of Sox9 and Dmrt2 in growth plate chondrocytes.

Sections of an E15.5 mouse tibia were subjected to H&E staining and immunohistochemical analyses using anti-Sox9 (green) and anti-Dmrt2 (red) antibodies. Higher-magnification images of the area boxed in (a) are shown in (b). Scale bars: (a) 250 μm and (b) 1000 μm.

Supplementary Figure 6

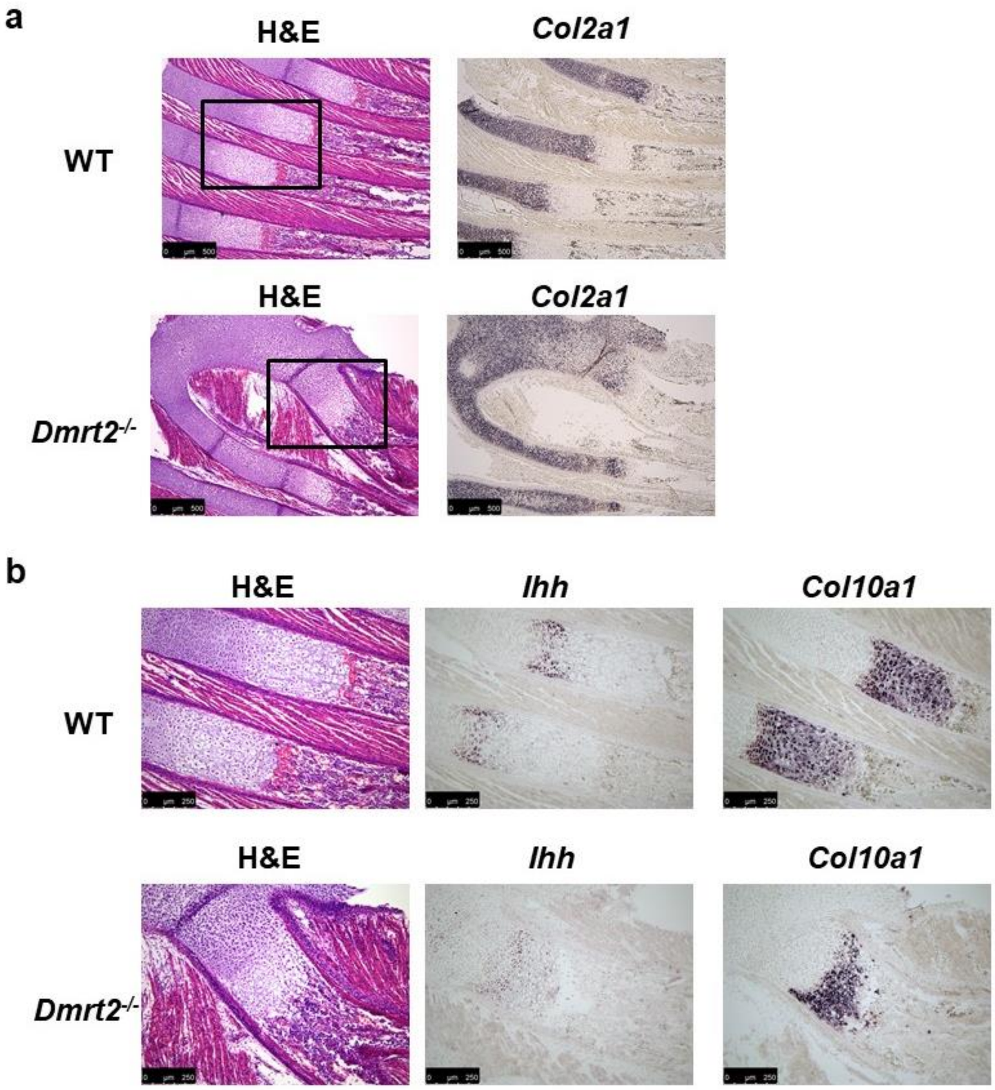

Supplementary Figure 6. *In situ* hybridization analysis of newborn mouse rib cartilage. Sections of rib cartilage from newborn WT and *Dmrt2*$^{-/-}$ littermates were examined by hematoxylin and eosin (H&E) staining and RNA *in situ* hybridization analysis using antisense probes against *Col2a1*, *Ihh*, and *Col10a1*. Higher-magnification images of the boxed area in (a) are shown in (b). Scale bars: (a) 500 μm and (b) 250 μm.

Supplementary Figure 7

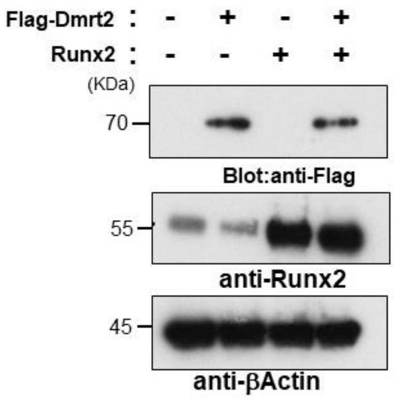

Supplementary Figure 7. Primary chondrocytes from WT mice were infected with Flag-Dmart2 and Runx2 adenovirus and cultured for 4 days. Cell lysates were analyzed using immunoblotting with anti-Flag, anti-Runx2, and anti-β-actin antibodies.

Supplementary Figure 8

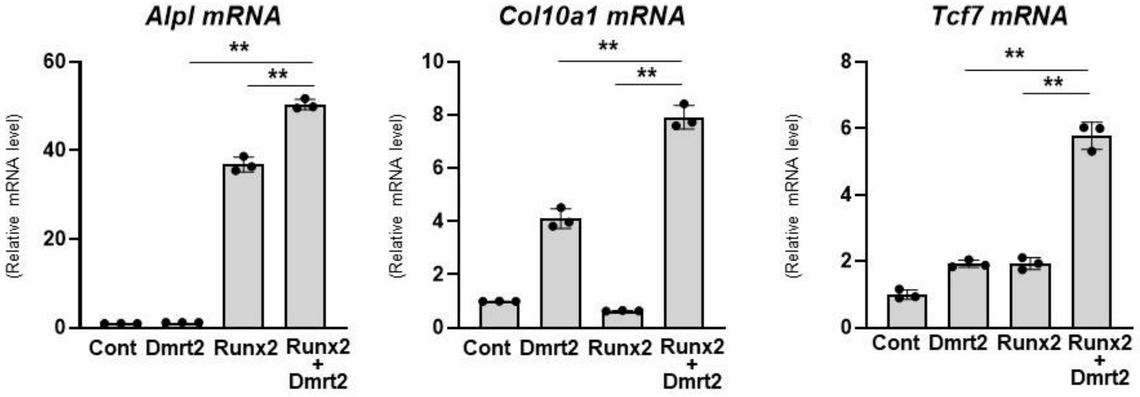

Supplementary Figure 8. Synergistic effect of Dmrt2 and Runx2 on hypertrophic chondrocyte-specific gene expression.

Dmrt2 and Runx2 were overexpressed in primary chondrocytes using adenoviruses, and then *Alpl, Col10a1*, and *Tcf7* mRNA expression levels were analyzed by RT-qPCR. Data are shown as the mean ± s.d. (n = 3). *p < 0.01; one-way ANOVA followed by the Tukey test.

Supplementary Figure 9

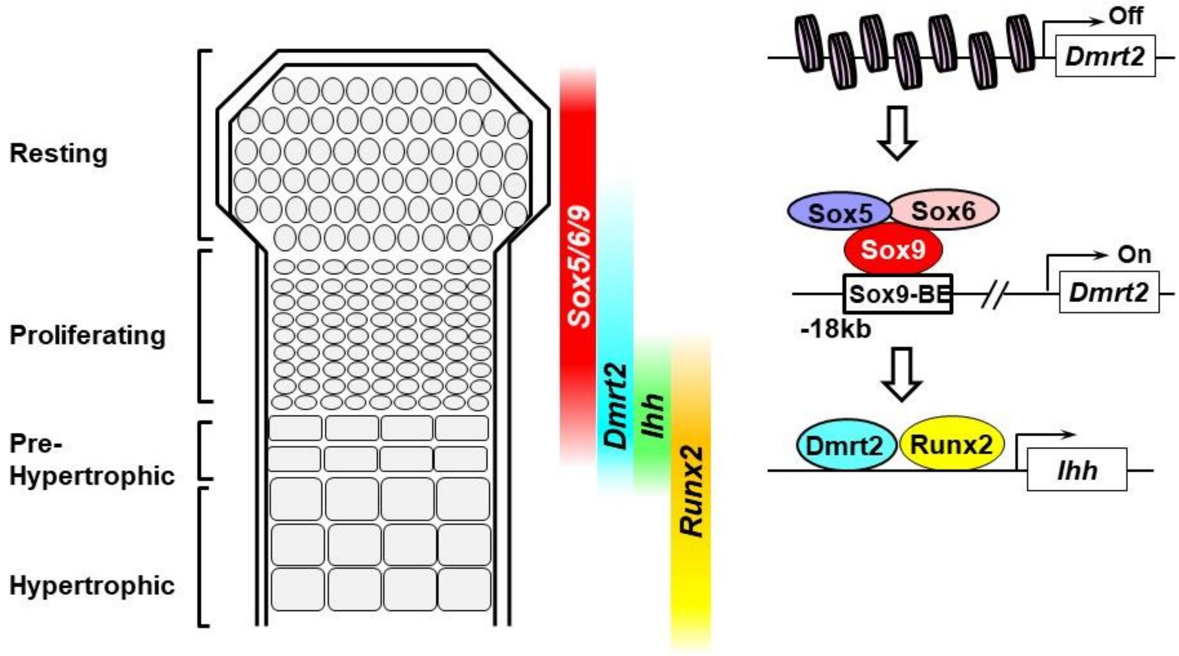

Supplementary Figure 9. A model depicting the role of Dmrt2 during endochondral bone formation.

Sox9 binding enhancer region of the Dmrt2 gene is closed and inactivated in the early stage of chondrocyte differentiation (upper panel). This enhancer region becomes open and epigenetically active according to chondrocyte differentiation, which allows Sox9 to bind to this enhancer with Sox5 and Sox6, thereby promoting Dmrt2 expression in pre-hypertrophic chondrocytes (middle panel). Subsequently, Dmrt2 augments *Ihh* gene expression through physical and functional interaction with Runx2 (lower panel).

Supplementary Figure 10

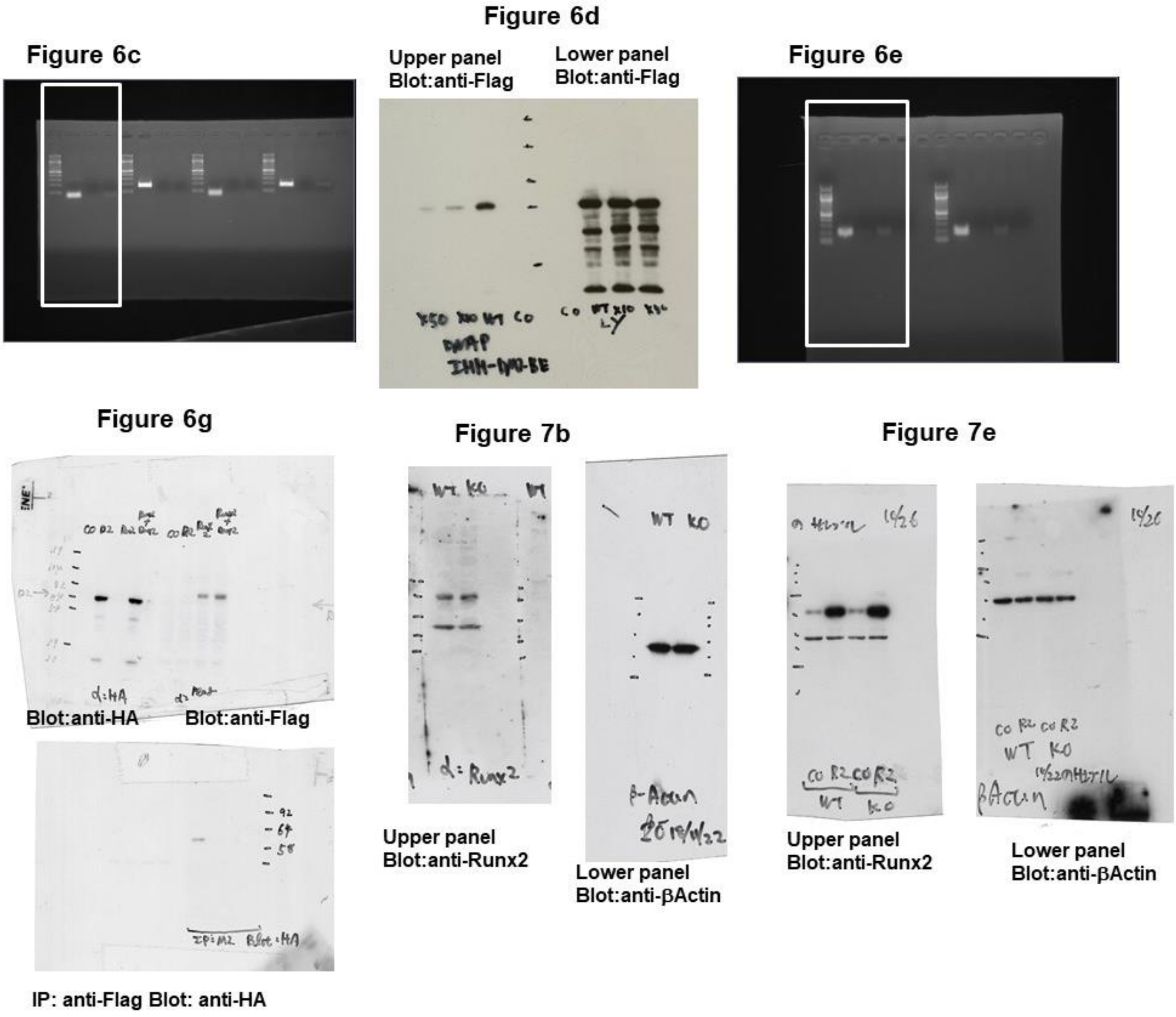

Supplementary Figure 10. Uncropped blot/gel images (Figure 6 and Figure 7)

Supplementary Figure 10 (continued)

Supplementary Figure 3a

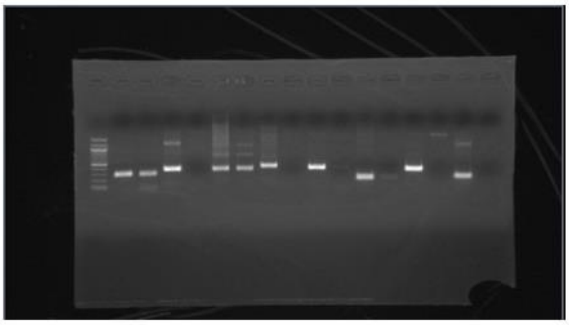

Supplementary Figure 3b

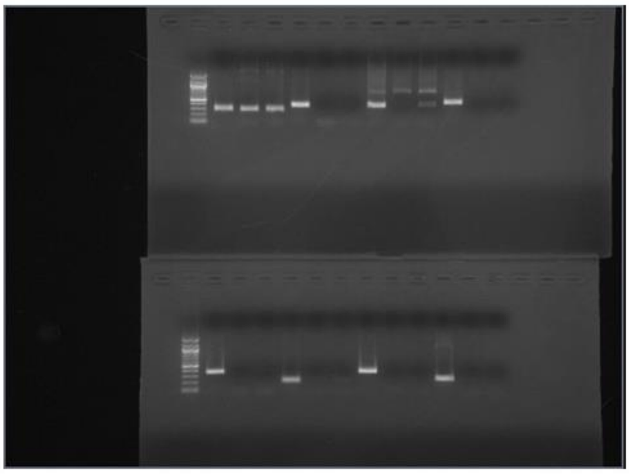

Supplementary Figure 7

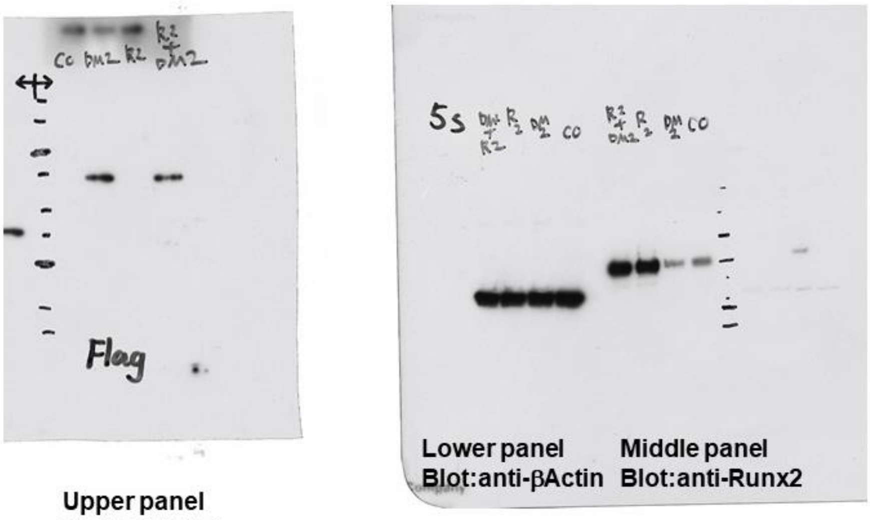

Upper panel
Blot:anti-Flag

Supplementary Figure 10. Uncropped blot/gel images (Supplementary Figure 3 and Supplementary Figure 7)
