# Supplementary material for: Dmrt2 promotes transition of endochondral bone formation by linking Sox9 and Runx2
Source: Commun Biol. 2021 Mar 11;4:326. doi: 10.1038/s42003-021-01848-1 (PMC7952723; doi:10.1038/s42003-021-01848-1)
Supplement: Supplementary file 2 — Description of Additional Supplementary Files [file 42003_2021_1848_MOESM2_ESM.pdf]

Description of Additional Supplementary Files

File Name: Supplementary Data 1

Description: All source data underlying Figures and Supplementary Figures.
